# Ethnoveterinary herbal remedies used by farmers in four north-eastern Swiss cantons (St. Gallen, Thurgau, Appenzell Innerrhoden and Appenzell Ausserrhoden)

**DOI:** 10.1186/1746-4269-10-32

**Published:** 2014-03-31

**Authors:** Monika Disler, Silvia Ivemeyer, Matthias Hamburger, Christian R Vogl, Anja Tesic, Franziska Klarer, Beat Meier, Michael Walkenhorst

**Affiliations:** 1 Institute of Pharmaceutical Biology, Departement of Pharmaceutical Sciences, University of Basel, Basel, Switzerland; 2 Departement of Livestock Science, Research Institute of Organic Agriculture, Ackerstrasse 113, Postfach, CH-5070 Frick, Switzerland; 3 Division of Organic Farming, Department of Sustainable Agricultural Systems, University of Natural Resources and Life Sciences (BOKU), Vienna, Austria; 4 Swiss Federal Institute of Technology Zurich, Zurich, Switzerland; 5 Unit of Phytopharmacy, Institute of Biotechnology, Zurich University of Applied Sciences, Wädenswil, Switzerland

**Keywords:** Ethnoveterinary, Herbal remedies, Switzerland (St. Gallen, Thurgau, Appenzell Innerrhoden, Appenzell Ausserrhoden), Farmers

## Abstract

**Background:**

Very few ethnoveterinary surveys have been conducted in central Europe. However, traditional knowledge on the use of medicinal plants might be an option for future concepts in treatment of livestock diseases. Therefore the aim of this study was to document and analyse the traditional knowledge and use of homemade herbal remedies for livestock by farmers in four Swiss cantons.

**Methods:**

Research was conducted in 2012. Fifty farmers on 38 farms were interviewed with the aid of semistructured interviews. Detailed information about the plants used and their mode of preparation were documented as well as dosage, route of administration, category of use, origin of knowledge, frequency of use, and satisfaction with the treatment.

**Results:**

In total, 490 homemade remedies were collected. Out of these, 315 homemade remedies contained only one plant species (homemade single species herbal remedies, HSHR), which are presented in this paper. Seventy six species from 44 botanical families were mentioned. The most HSHR were quoted for the families of *Asteraceae*, *Polygonaceae* and *Urticaceae*. The plant species with the highest number of HSHRs were *Matricaria recutita* L*.*, *Calendula officinalis* L*.*, *Rumex obtusifolius* L*.* and *Urtica dioica* L. For each HSHR, one to eight different applications were enumerated. A total of 428 applications were documented, the majority of which were used to treat cattle. The main applications were in treatment of skin afflictions and sores, followed by gastrointestinal disorders and metabolic dysfunctions. Topical administration was most frequently used, followed by oral administration. In nearly half of the cases the knowledge on preparing and using herbal remedies was from forefathers and relatives. More than one third of the applications were used more than ten times during the last five years, and in about sixty percent of the cases, the last application was during the last year preceding the interviews.

**Conclusions:**

Traditional knowledge of farmers about the use of medicinal plants to treat livestock exists in north-eastern Switzerland. Homemade herbal remedies based on this knowledge are being used. The interviewed farmers were satisfied with the outcome of the applications.

## Abstract (de)

**Zusammenfassung:**

**Hintergrund**

Aus weiten Teilen Mitteleuropas fehlen bisher Angaben zur Ethnoveterinärmedizin. Andererseits birgt das traditionelle Erfahrungswissen zu Pflanzen und deren Anwendungen ein grosses Potential für zukünftige Behandlungsstrategien für Nutztierkrankheiten. Ziel dieser Studie war daher die Erfassung und Auswertung des traditionellen Erfahrungswissens zu pflanzlichen Hausmitteln und deren Anwendungen bei Nutztieren auf landwirtschaftlichen Betrieben in vier nordöstlichen Schweizer Kantonen (St. Gallen, Thurgau, Appenzell Innerrhoden und Appenzell Ausserrhoden).

**Methoden**

Von Anfang März bis Ende April 2012 wurden 50 Landwirtinnen und Landwirte auf 38 Betrieben anhand eines semistrukturierten Fragenkatalogs interviewt. Für jede genannte Rezeptur wurden detaillierte Informationen zu den verwendeten Pflanzen und zur Herstellung dokumentiert, darüber hinaus für deren Anwendungsgebiete die Dosierung, Verabreichungsart, Anwendung, Wissensursprung, Einsatzhäufigkeit und die Zufriedenheit mit dem Ergebnis der Therapie.

**Resultate**

Insgesamt wurden 490 Rezepturen erfasst. Davon enthielten 315 Rezepturen je nur eine einzelne Pflanzenart. Ausschliesslich diese Rezepturen wurden in die Auswertung einbezogen. Sechsundsiebzig verschiedene Pflanzenarten aus 44 Pflanzenfamilien wurden hierfür verwendet. Die Pflanzenfamilien *Asteraceae*, *Polygonaceae* und *Urticaceae* waren am häufigsten vertreten. Die meistgenannten Pflanzenarten waren *Matricaria recutita* L*.*, *Calendula officinalis* L., *Rumex obtusifolius* L*.* und *Urtica dioica* L. Für jede Rezeptur wurden bis zu acht verschiedene Anwendungen, insgesamt 428 überwiegend für Rinder, beschrieben. Die meistgenannten Anwendungsgebiete waren Hautveränderungen und Wunden, sowie Erkrankungen des Magen-Darm-Traktes und Stoffwechselstörungen. Am häufigsten wurden topische Verabreichungen genannt, gefolgt von den oralen Verabreichungen. Das Wissen über die Herstellung und Verwendung der Rezepturen stammt fast zur Hälfte von Vorfahren und Verwandten. Mehr als ein Drittel der Anwendungen wurde während der letzten fünf Jahre zehnmal und häufiger benutzt. Die letzte Anwendung erfolgte in rund sechzig Prozent der Fälle innerhalb der letzten zwölf Monate.

**Schlussfolgerung**

Das traditionelle Erfahrungswissen über pflanzliche Hausmittel und deren Anwendungen bei Nutztieren ist in der nordöstlichen Schweiz vorhanden. Die auf diesem Erfahrungswissen basierenden pflanzlichen Rezepturen werden nach wie vor angewendet. Die pflanzlichen Rezepturen werden mit grosser Zufriedenheit zur Behandlung von Nutztieren eingesetzt.

## Background

Ethnoveterinary research is defined as the “systematic investigation and application of veterinary folk knowledge, theory and practise” [1]. In recent years, ethnoveterinary studies have been conducted mainly in Africa, Asia, and in Central America [2]. In developing countries, animal health care is often based on the use of self-made preparations, particularly when access to western veterinary products is difficult or too expensive for the local farmer [3]. Few ethnoveterinary studies on herbal remedies have been conducted in Europe, and surveys have been published for Spain [4-7], the overall mediterranian region [8], Italy [9-11] and Austria [12-15]. Two ethnoveterinary studies have been recently carried out in Switzerland, one in three cantons of the central-northern part [16], and the second in a valley (Safiental) of the canton of Graubünden [17].

Traditional medicine is defined by the World Health Organization as “the sum total of the knowledge, skills, and practices based on the theories, beliefs and experiences indigenous to different cultures, whether explicable or not, used in the maintenance of health as well as in the prevention, diagnosis, improvement or treatment of physical and mental illness” [18]. Other authors specify a tradition as the transmission of knowledge over at least three generations, whereas the life span of a generation is not generally defined and varies widely [19]. The traditional knowledge of use of medicinal plants which was transmitted from generation to generation is recently in imminent danger of disappearing [20]. Hence, traditional knowledge might be an option for future concepts in treatment of livestock diseases and, for this reason, should be documented before it gets lost.

European Council and Swiss regulations of organic agriculture emphasize the use of “phytotherapeutic products”, “homeopathy”, and “micronutrients” for the treatment of livestock diseases. “Chemically synthesised allopathic veterinary medicinal products including antibiotics” may be used where necessary, but only within strict limitations [21,22]. A total of eleven veterinary medicinal products with herbal ingredients are currently available in Switzerland [23], among these only three products with pure plant ingredients are available [24]. Only few years ago a substantially higher number of herbal veterinary medicinal products were available [23]. Hence, organic farmers have few other choices than using their own herbal remedies.

The aim of this research was to document the veterinary usage of plants by farmers in the four neighbouring Swiss cantons of St. Gallen, Thurgau, Appenzell Innerrhoden and Appenzell Ausserrhoden. The focus was on establishing a list of medicinal plants used in homemade remedies, on the preparation of remedies, their uses, and estimation of the amounts of medicinal plant in the final product. The results were compared with other ethnoveterinary studies that have been conducted in Switzerland and in Europe.

## Methods

The methodology of the study was according to the previous project ‘Traditional use of herbal remedies in livestock by organic farmers in three Swiss cantons (Aargau, Zurich, Schaffhausen)” [16].

### Study area

The study area included the four cantons of St. Gallen, Thurgau, Appenzell Innerrhoden and Appenzell Ausserrhoden, which are situated in the north-east of Switzerland. The four neighbouring cantons are in part adjacent to the region covered by the previous project [16]. The research area is located between 8°4’ and 9°4’ E and 46°5’ and 47°4’ N, and the altitude varies between 370 m and 3247 m above sea level [25]. At a mean altitude of 595 m above sea level the average annual temperature is 7.9° Celsius. The average annual precipitation ranges between 1000 and 1900 millimetres [26,27]. The research area covers a region of 3432 km^2^, and has approximately 800’000 inhabitants [28].

There were a total of 8887 farms in the four cantons. In St. Gallen 386 (8.4%) out of 4592 farms, in Thurgau 241 (8.2%) out of 2947, in Appenzell Innerrhoden 22 (4.2%) out of 534, and in Appenzell Ausserrhoden 111 (13.6%) out of 814 were organic farms. As the most frequently animal species 6528 (73.5%) out of those 8887 farms kept cattle, 4254 (47.9%) poultry, and 1606 (18.1%) kept pigs [29].

### Dialogue partners

**Figure 1:**
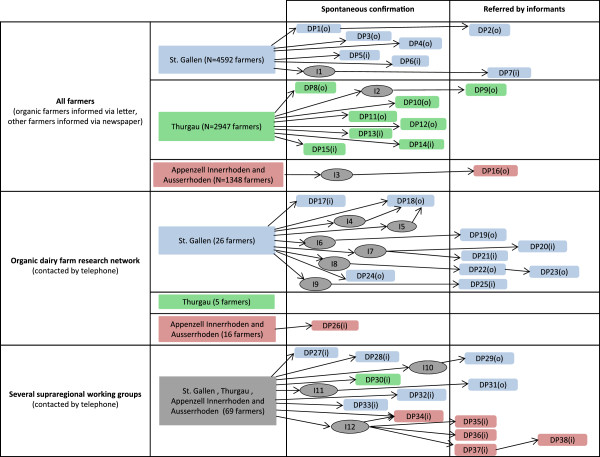
**Snowball sampling.** DP = dialogue partners; blue = DP from St. Gallen; green = DP from Thurgau; red = DP from Appenzell Innerrhoden and Appenzell Ausserrhoden; grey = supraregional working groups and informants; I = informants; (i) = integrated production (non-organic farms); (o) = organic farms; N = all farmers operating in this canton.

Different methods were used to recruit dialogue partners. In a first step a letter with detailed information about the project was sent to all organic farmers in the research area. A broader population was informed about the research through publications in the local agricultural press. Furthermore, the project was presented at two farmers’ meetings on complementary medicine. Persons contacted were asked to support the project, either as dialogue partners, or as informants (without own knowledge regarding herbal remedies) providing information on other farmers according to the snowball sampling method [30]. The contacts of the informants are leading to further dialogue partners (Figure 1).

Twelve farmers spontaneously agreed to become dialogue partners. All farmers in the study area that belonged to the organic dairy farm research network of the Research Institute of Organic Agriculture in Frick were contacted by phone and asked to support the project. Four additional dialogue partners were recruited by this contact. In addition, all 69 farmers of several supraregional working groups on complementary medicine in the study area were invited by phone. Six farmers could be recruited by this approach. Finally, snowball sampling [30] through several informants led to recruitment of additional 16 interview partners (Figure 1).

The following criteria had to be fulfilled by the farmers to qualify as a dialog partner:

– The farm had to be in the research area.

– The dialogue partner had to nominate at least three different applications of homemade remedies or medical plants.

– The dialogue partner had to agree sharing his/her knowledge to the research team, for analysis and publication of the data in an anonymized form.

A total of 38 interviews were conducted between the beginning of March and the end of April 2012. In most cases dialogue partners were interviewed alone. On nine farms one and on one farm three further persons (all family members) asissts the dialogue partner for the interview. As a consequence the information comes from 50 farmers, or members of the farmers’ family. The answers given by persons assisting the dialogue partner were added to the data of the dialogue partner and not analysed separately. For example, if during an interview a member of the farmer’s family named a homemade remedy, which was applied on the same farm by him/her, the answers were combined with the interview of the main dialog partner.

A total of 29 women (58%) and 21 men (42%) with an age between 30 and 81 (55 ± 13) years served as dialogue partners or assisting persons. Twenty-two interviews were held in the canton of St. Gallen, nine in Thurgau, two in Appenzell Innerrhoden, and five in Appenzell Ausserrhoden. All farmers, but also the persons assisting the main dialogue partner, were active in animal care on at least one farm. This could be their own farm or, for example, the farm of their descendants. In addition, some of dialogue partners provided their advice to other farmers, or they served as herdsmen on alpine pasture holdings during the summer months.

### Farms

The altitude of the location of the farms in the research area varied considerably, as the 38 farms were located between 440 m and 1200 m above sea level. Fourteen farms were below 600 m, 13 between 600 m and 900 m, and eleven on an altitude above 900 m. The sample comprised 17 organic and 21 non-organic farms. All farms kept cattle. Thirty six were dairy farms and two were suckler cow husbandries. In addition, 13 farms kept hens, twelve farms had pigs, twelve farms goats, eight farms sheep and five farms horses. The dairy farms kept between seven and 40 cows, the suckler cow farms between 16 and 21 cows. On three farms the agricultural area was between six and ten hectars, on eleven farms between eleven and 15 hectares, on 16 farms between 16 and 25 hectares, and on eight farms the agricultural area was more than 25 hectares.

### Data collection and analysis

The dialogue partners were asked to give a written agreement for recording the interview and to use the data for analysis and for publication in an anonymized form. The records were not transcribed, but secured for later reference (recorded by OLYMPUS WS 200S Digital Voice Recorder, Olympus Imaging Europa GmbH, Hamburg, Germany). During the face-to-face interviews, the answers were noted on questionnaire forms, and complemented with the audio records. Final data were entered into a database [31].

The interviews were subdivided into three parts: 1) general information about the farm; 2) a semi-structured part constisting of seven “free listing” questions [32]; 3) a structured part with pre-coded and free answer questions to gain detailed information about the specific homemade remedies and their uses. The duration of the interviews was between 1.5 and 4.0 hours.

After documentation of some general structural data of the farm like livestock species or farm size (part one of the interview), first impressions of the farmers’ knowledge about plants and formulations were collected in the “free listing” part (part two of the interview). This part of the interview also served to create a casual and pleasant athmosphere. The third part of the interview focused on specific homemade remedies. Depending on the particular question the respondent had pre-coded and free answer possibilities. Plants collected from the wild or cultivated in home gardens were identified with the aid of the standard taxonomic reference “Flora Helvetica” [33]. Herbal drugs and extracts from commercial sources were identified with the aid of their package leaflet, or were assumed to be correctly delivered by the pharmacies. Whenever possible, a photographic documentation of the dried plants or their more or less processed products was made. It was not possible to collect herbarium voucher specimens during the interviews, since they were conducted in early spring. However, it was possible to collect from July to September 2012 a total of 16 herbarium voucher specimens on 10 farms. These specimens included 10 plant species collected in the wild. Herbarium voucher specimens were dried, labelled and deposited at the botanical repository of the University of Zurich and the Swiss Federal Institute of Technology Zurich (Botanischer Garten, Zollikerstrasse 107, 8008 Zurich, Switzerland).

The dialogue partners were asked for information on the manufacturing processes for their homemade herbal remedies. Details such as source of the herbal material, and procedures for extraction and preparation of the finished product were documented. Whenever possible, the amount of plant used was determined on site with the aid of a scale (Mettler P1000N, Mettler- Toledo GmbH, Greifensee, Switzerland), in order to calculate concentrations in g dry plant equivalent per 100 g of finished product. This could be conducted either with plants from the dialog partner, or with a collection of herbal material of Pharmacopoeia quality [34] purchased by pharmacy. If this was not possible, dosages were estimated by assessment of the administered volume of a herb and subsequent weighing.

The reported uses for each remedy were recorded. The respondent could give a free answer which was afterwards coded into categories of use according to the anatomical therapeutic chemical classification system for veterinary medicinal products ATCvet [35]. Route and frequency of administration, and mean duration of treatment were also recorded. The routes of administration were classified into external, internal, and treatment of housing environment. External administration was defined as administration onto intact skin, and altereted or sore skin, respectively. If the preparation was administered into a body orifice (oral, intravaginal/intrauterine administration, inhalation) it was classified as internal administration. Treatment of environment or stable was defined as treatment to improve animal health, but without direct contact to the animal itself.

The daily dosage of medicinal plant (dry plant equivalent) was calculated for all prepratations that were administered orally. For a comparison between different species (including human), daily dosages were normalized by a conversion of all dosages into dosage per kilogram metabolic bodyweight (MBW = bodyweight^0.75^) [36]. The following formula was used:

(1) $$\mathrm{daily}\;\mathrm{dose}\;\left(\frac{g}{kg^{0.75}}\right)=\frac{\mathrm{drug}\;\mathrm{dose}\;\mathrm{per}\;\mathrm{administration}\;\left(g\right)\times \mathrm{repetition}\;\mathrm{per}\;\mathrm{day}}{\mathrm{metabolic}\;\mathrm{bodyweight}\;\left(kg^{0.75}\right)}$$

Live weight of animals were taken from Table 1[37,38].

**Table 1:** **Metabolic body weight of different species based on estimated average body weights**[37,38]

**Species**	**Weight**	**Metabolic bodyweight (MBW)**
Adult cattle	650 kg	128.7 kg^0.75^
Calf	75 kg	25.5 kg^0.75^
Pig	200 kg	53.2 kg^0.75^
Young pig	15 kg	7.6 kg^0.75^
Donkey	200 kg	53.2 kg^0.75^
Goat	50 kg	18.8 kg^0.75^
Medium-sized dog	25 kg	11.2 kg^0.75^
Young sheep	20 kg	9.5 kg^0.75^
Rabbit	3 kg	2.3 kg^0.75^
Hen	1 kg	1.0 kg^0.75^
Rat	0.175 kg	0.3 kg^0.75^
Human	65 kg	22.9 kg^0.75^

For topically administered preparations, the concentration in g drug equivalent per 100 g of finished product was calculated.

Furthermore, the origin of knowledge for homemade remedies was documented, as well as the interview partners’ estimate on the frequency of use during the last five years, and the date of the last use. Also, information whether remedies were administered solely or in combination with other therapies was recorded.

To evaluate the satisfaction with the outcome of the application, a visual analog scale (VAS) was used [39]. A scale of 100 mm was used, with “no effect” corresponding to 0 mm, and “very good effect” to 100 mm. Mean and standard deviation of the VAS were calculated for each category of use.

In a second phase of the study, data were compared with the results from earlier ethnoveterinary studies in other parts of Switzerland [16,17], in Austria [12-15], Italy [2,9,11], and in Spain [4-7].

### Definitions

#### Homemade remedy

We defined as one homemade remedy a description of a preparation from one dialogue partner containing one ore more plants, plant parts or other natural compounds more or less processed to a finished product: [dialogue partner] × [plant species or other natural compounds] × [plant part] × [manufacturing process to the finished product].

#### Application

We sampled only homemade remedies which were intended to be administered to an animal in case of a disease or as a preventive measure. Therefore one homemade remedy was connected to one or more applications. We defined as one application the description of the use of one homemade remedy as combination of the category of use (for example skin alterations and sores), the specification of use (for example an open wound or a skin infection), the intended animal species, the age classification of the intended animal and the administration procedure: [homemade remedy] × [category of use] × [specification of use] × [animal species] × [animal age classification] × [administration procedure].

## Results

The dialog partners listed between four and 28 homemade remedies each (mean 12.9 ± 5.4) and mentioned between one and eight different applications for each homemade remedy (mean 1.3 ± 0.7). This led to a total of 490 homemade remedies. Ninety-four plant species belonging to 50 plant families were mentioned. Thirty-two out of the 490 homemade remedies included homemade complex herbal remedies containing two to nine plant species, 143 homemade remedies without plant species but containing one or more natural products like curds, eggs, honey, lard, pure alcohol, propolis, red wine, salt, soft soap or vinegar, and 315 homemade remedies (including 19 based on commercial extracts) containg only one plant species (homemade single species herbal remedies, HSHR).

### Composition and manufacturing process of the 315 HSHR

Only the 315 homemade HSHR (see Additional file 1) were analysed in detail. They contained 67 different plant species belonging to 44 families. Plants belonging to the *Asteraceae* were the most frequently reported uses (73 HSHR, 23.2%), followed by *Polygonaceae* (21 HSHR, 6.7%) and *Urticaceae* (21 HSHR, 6.7%). The species with the highest number of reports were *Matricaria recutita* L. (26 HSHR, 8.3%), *Calendula officinalis* L*.* (24 HSHR, 7.6%), *Rumex obtusifolius* L. (21 HSHR, 6.7%), and *Urtica dioica* L. (21 HSHR, 6.7%) (Table 2).

**Table 2:** Extraction procedure to prepare the 315 homemade single species herbal remedies (HSHR)

**Botanical family**	**Plant species with ≥ 3 named HSHR**	**On farm extraction procedure (Numbers indicate the frequency of mentioned 315 HSHR)**						
**(Number of named plant species in this family)**	**(Numbers indicate the frequency of mentioned 315 HSHR)**							
		**None**	**Water**			**Alcohol**	**Oil/Fat**	
			**Room temperature**	**Infusion**	**Decoction**	**Room temperature**	**Room temperature**	**Heated up**
**Asteraceae (11)**	All asteraceae (73)	11		33		12	13	4
	***Matricaria recutita *L.** (26) **[vs]**	2^a^		22			2	
	Flos (26)							
	***Calendula officinalis *L.** (24)	4^b^		2		6	8	4
	Flos and flos sine calice (24)							
	***Arnica montana *L.** (8)	2^c^				5	1	
	Flos (8)							
	***Senecio ovatus *Willd.** (4)			4				
	Herba (4)							
	***Senecio alpinus *(L.) Scop** (3) **[vs]**			3				
	Herba (3)							
	Others^1^ (8)	3^d^		2		1	2	
**Polygonaceae (1)**	***Rumex obtusifolius *L.** (21) **[vs]**				4			
	Radix (4)							
	Folium (16)	3					6	7
	Herba cum radice (1)			1				
**Urticaceae (1)**	***Urtica dioica *L.** (21) **[vs]**	11		10				
	Herba (21)							
**Malvaceae (3)**	All Malvaceae (19)	1	1	10	4		2	1
	***Malva neglecta *Wallr**. (13) **[vs]**		1	8			2	1
	Herba (12)							
	Flos (1)			1				
	***Tilia cordata Mill.*** (5)	1			4			
	Cortex (5)							
	Others^2^ (1)			1				
**Rubiaceae (1)**	***Coffea arabica *L.** (16)			16				
	Semen (16)							
**Boraginaceae (1)**	***Symphytum officinale *L.** (15) **[vs]**	5^e^				4	2	2
	Radix (13)							
	Folium (2)	2						
**Rosaceae (7)**	All Rosaceae (15)	11		1	2	1		
	***Crataegus laevigata *(Poir.) DC*.*** (4)	4						
	Herba (4)							
	***Potentilla erecta *(L.) Raeusch.** (4) **[vs]**	1^f^			2	1		
	Rhizoma (4)							
	***Prunus spinosa *L.** (3)	3						
	Herba (3)							
	Others^3^ (4)	3		1				
**Pinaceae (2)**	All Pinaceae (13)	7						6
	***Picea abies *(L.) H**. **Karst**. (12)	5						
	Herba (5)							
	Resina (7)	1						6
	Others^4^ (1)	1						
**Hypericaceae (1)**	***Hypericum perforatum *L.** (12) **[vs]** Flos (12)	2^g^					10	
**Linaceae (1)**	***Linum usitatissimum *L.** (11)	2			9			
	Semen (11)							
**Lamiaceae (4)**	All Lamiaceae (9)	6		2			1	
	***Mentha canadensis *L.** (3)	2^h^		1				
	Folium (3)							
	***Salvia officinalis *L.** (3)	2		1				
	Folium (3)							
	Others^5^ (3)	2^i^					1	
**Apiaceae (4)**	All Apiaceae (8)			5	2	1		
	***Carum carvi *** **L. (3)**							
	Fructus (3)			1	2			
	***Sanicula europaea *** **L. (3)**							
	Herba (3)			2		1		
	Others^6^ (2)			2				
**Brassicaceae (3)**	All Brassicaceae (8)	7	1					
	***Brassica oleracea *** **L. (4)**							
	Folium (4)	4						
	***Brassica napus *** **L. (3)**							
	Oleum (3)	3						
	Others^7^ (1)		1					
**Geraniaceae (2)**	All Geraniaceae(8)	3				1	3	1
	***Geranium robertianum *** **L.s.str. (7) [vs]**							
	Herba (7)	2				1	3	1
	Others^8^ (1)	1^k^						
**Amaryllidaceae (2)**	All Liliaceae (6)	4	2					
	***Allium sativum *** **L. (4)**							
	Bulbus (4)	2	2§					
	Others^9^ (2)	2						
**Theaceae (1)**	***Camellia sinensis *** **(L.)** **Kuntze (6)**							
	Folium (6)			6				
**Adoxaceae (1)**	***Sambucus nigra *** **L. (5)**							
	Herba (5)	4		1				
**Fagaceae (1)**	***Quercus robur *** **L. (5)**							
	Cortex (5)	1			4			
**Aquifoliaceae (1)**	***Ilex aquifolium *** **L. (4)**							
	Herba (3)	3						
	Folium (1)				1			
**Poaceae (1)**	***Avena sativa *** **L.s.str. (3)**							
	Fructus (3)	1			2			
**Rhamnaceae (1)**	***Rhamnus cathartica *** **L. (3)**							
	Herba (3)	3						
**Others**^**10 **^**(26)**	**26 other plant species (34) [vs]**	18^1^		7	2	3	3	1
**Total (76)**		**105**	**4**	**92**	**30**	**22**	**40**	**22**

**[vs]:** voucher specimens available, voucher numbers are visible in the Additional file 1.

^1^*Arnica chamissonis* Less. (2), *Solidago virgaurea* L. (2), *Achillea millefolium* L. (1), *Helianthus annuus* L. (1), *Tanacetum vulgare* L. (1), *Tanacetum parthenium* (L.) Sch. Bip. (1); ^2^*Althaea officinalis* L. (1); ^3^*Alchemilla vulgaris* L.Agg. (1), *Rubus idaeus* L. (1), *Malus domestica* Borkh. (1), *Prunus domestica* L. (1); ^4^*Abies alba* Mill. (1); ^5^*Lavandula angustifolia* Mill. (2), *Thymus vulgaris* L. (1); ^6^*Petroselinum crispum* (Mill.) Fuss (1), *Foeniculum vulgare* Mill. (1); ^7^*Capsella bursa- pastoris* (L.) Medik (1); ^8^*Pelargonium sidoides* DC (1); ^9^*Allium cepa* L. (2); ^10^*Beta vulgaris subsp. vulgaris* Conditiva group (1) (Amaranthaceae); *Dryopteris filix-mas* (L.) Schott (1) (Aspidiaceae); *Betula pendula* Roth (1) (Betulaceae); *Cannabis sativa* L. (1) (Cannabaceae); *Chenopodium bonus-henricus* L. (1) (Chenopodiaceae); *Juniperus communis* L.s.str. (2) (Cupressaceae); *Thuja occidentalis* L. (2) (Cupressaceae); *Equisetum arvense* L. (1) (Equisetaceae); *Vaccinium myrtillus* L. (2) (Ericaceae); *Anthyllis vulneraria* L.s.str. (1) (Fabaceae); *Gentiana lutea* L. (1) (Gentianaceae); *Juglans regia* L. (1) (Juglandaceae); *Cinnamomum verum* J.Presl (1) (Lauraceae); *Lycopodium clavatum* L. (1) (Lycopodiaceae); *Myristica fragrans* Houtt. (2) (Myristicaceae); *Melaleuca alternifolia* Maiden&Betche ex Cheel (2) (Myrtaceae); *Fraxinus excelsior* L. (2), *Olea europaea* L. (1) (Oleaceae); *Pedicularis verticillata* L. (1) (Orobanchaceae); *Euphrasia officinalis* L. (1), *Plantago lanceolata* L. (2) (Plantaginaceae); *Citrus x limon* (L.) Burm.f. (1) (Rutaceae); *Salix caprea* L. (1) (Salicaceae); *Quassia amara* L. (1) (Simaroubaceae); *Solanum tuberosum* L. (2) (Solanaceae); *Tropaeolum majus* L. (1) (Tropaeolaceae).

^a^Kamillosan® Liquidum used in two remedies; ^b^Calendula tincture (pharmacy) used in one remedy; Calendula ointment (pharmacy) used in three remedies; ^c^Arnica oil (pharmacy) used in one remedy; Arnica ointment (pharmacy) used in one remedy; ^d^Goldenrod ointment (pharmacy) used in one remedy; ^e^Comfrey emulsion (pharmacy) used in one remedy; ^f^Common tormentil tincture (pharmacy) used in one remedy; ^g^St John's wort oil (pharmacy) used in two remedies; ^h^NPJ Liniment® and OPIFIX® (both containing Mentha arvensis L. var. piperascens) used in two remedies; ^i^Thyme oil used in one remedy; ^k^Pelargonium Spray® used in one remedy; ^l^Tea Tree oil used in two remedies.

§ one extraction with milk.

The most commonly used plant parts were flowers (75 HSHR, 23.8%). Herbs (aerial parts of a herbaceous plant, without roots) were used in 66 HSHR (21.0%), followed by fruits, seeds and berries (45 HSHR, 14.3%) and leaves (41 HSHR, 13.0%). Other plant parts, such as twigs, roots, barks, whole plants, and plant excretions such as resins were mentioned (Table 2). In 150 HSHR (47.6%) wild-harvested plants were used, while commercial drugs and cultivated plants were used in 97 HSHR (30.8%) and 68 HSHR (21.6%), respectively.

Fresh plants were used in 58.4% HSHR, and dried plants in 35.6% of the HSHR. In 19 HSHR (6.0%), commercial products, such as Kamillosan® (MEDA Pharma GmbH, Wangen-Brüttisellen; Switzerland), NPJ Liniment® (Casa Verde GmbH, Dortmund, Germany) and OPIFIX® (Multiforsa AG, Auw, Switzerland) (both containing *Mentha canadensis* L.), Pelargonium Spray (containing *Pelargonium sidoides* DC; Alpinamed AG, Freidorf, Switzerland), and various products manufactured and marketed by local pharmacies (eg. tea tree oil, thyme oil, and various products of arnica, calendula, comfrey, common tormentill, goldenrod, and St John’s wort oil) were used. Preparations containing one of these commercial products are assigned to the remedies prepared without extraction (Table 2), because no extraction was carried out on the farm.

Beside these 19 commercial products (6.0%), additional 86 HSHR (27.3%) contained plants that were processed to the final remedy without any extraction step being performed. Such remedies served mainly for oral administration, topical treatment of injuries, or treatment of animal housing. Collard (*Brassica oleracea* L.), comfrey (*Symphytum officinale* L.), beetroot (*Beta vulgaris*), and broad-leaved dock (*Rumex obtusifolius* L.), for example, were directly applied onto intact skin for the treatment of injuries like inflamed joints. In 126 HSHR (40.0%) extraction was done with water (including one extraction with milk, containing garlic). These HSHRs were mainly for oral or external administration. Ninety two out of these 126 extracts were infusions, 30 decoctions, and four macerations (extraction at ambient temperature). In 62 HSHR (19.7%) the farmers used oil or fat as extracting agent, mainly for external administration. In 40 of these 62 HSHR extraction was carried out at ambient temperature, and in 22 cases elevated temperature was used. Maceration with alcohol was mentioned in 22 HSHR (7.0%) (Table 2).

A total of 49 HSHR (15.6%) were ointments, and all of them were prepared from fresh plant material. The plants used in these ointments included *Calendula officinalis* L. (flos; 11), *Rumex obtusifolius* L*.* (folium; 11), *Picea abies* (L.) H. Karst. (resina; 6), *Geranium robertianum* L.s.str. (herba; 4), *Symphytum officinale* L. (radix; 4), *Malva neglecta* Wallr. (herba; 3), *Arnica montana* L. (flos; 2), *Matricaria recutita* L. (flos; 2), *Solanum tuberosum* L. (potato peelings; 2), *Juniperus communis* L*.* (herba; 1), *Solidago virgaurea* L.s.str. (herba; 1), *Hypericum perforatum* L*.* (flos; 1) or *Chenopodium bonus-henricus* L. (folium; 1). The most frequently used ointment base was bees’ wax (38 HSHR). In six cases the ointment base (lard and milking grease, a kind of vaseline) served directly as extractant. Other animal fats and vegetable oils were also used as ointment base.

In 96 HSHR (30.5%) it was possible to estimate the amount of plant used in the remedies directly on the farm. This was done either with plant material provided by the interview partners (80 HSHR, 25.4%), or with the aid of plant samples from our collection of herbal drugs (16 HSHR, 5.1%). In 107 cases (34.0%) the weight was estimated by assessment of the administered volume of a plant and subsequent weighing. In additional 112 cases (35.5%) it was not possible to determine the weight of the plants used.

### Categories of use of the 428 applications of the 315 HSHR

In total, 428 applications (see Additional file 1) were mentioned for the 315 HSHR to treat cattle, goats, horses, pigs, rabbits, hens, donkeys, sheep and dogs. The most frequently reported uses were for treatment of skin alteration and sores (182 applications, 42.5%), gastrointestinal disorders and metabolic dysfunction (94 applications, 22.0%), treatment of the musculoskeletal system (incl. hematoma in the connective tissue) (37 applications, 8.6%), infertility and diseases of female genitals (36 applications, 8.4%), mastitis (18 applications, 4.2%), respiratory tract diseases (14 applications, 3.3%), and others (47 applications, 11.0%; treatment of parasitic diseases, behaviour and sensory organs, and general strengthening) (Table 3).

**Table 3:** 428 applications of 315 homemade herbal remedies containing a single herb (HSHR): routes of administration, categories of use, and target animal species

**Botanical family (Number of named plant species in this family)**	**Plant species with ≥ 3 named HSHR (Number of named remedies are given in brackets)**	**(Numbers indicate the frequency of mentioned applications, 428 applications are mentioned totally)**																
		**Routes of administration**						**Categories of use**							**Target animal species**			**Total different applications**
		**External**		**Internal**			**Treatment of housing environment**											
		**I**	**A**	**OR**	**IH**	**IU**		**Skin**	**Gast**	**Infe**	**Mast**	**Musc**	**Resp**	**Others**^**11**^	**Cattle**	**No spec.**	**Others**^**12**^	
**Asteraceae (11)**	All Asteraceae (73)	13	77	17		5		79	16	6	1	6		4	92	15	5	112
	***Matricaria recutita *** **L. (26)**		17^a^	13		4		15^a^	13	5				1	31^a^	2	1	34
	Flos (26)																	
	***Calendula officinalis *** **L. (24)**	4^b^	39^b^	1		1		42^b^	1	1		1			35^b^	7^b^	3	45
	Flos and flos sine calice (24)																	
	***Arnica montana *** **L. (8)**	5^c^	5					5				5^c^			6^c^	4		10
	Flos (8)																	
	***Senecio ovatus *** **Willd. (4)**		5					5							3	2		5
	Herba (4)																	
	***Senecio alpinus *** **(L.) Scop (3)**		3					3							3			3
	Herba (3)																	
	Others^1^ (8)	4^d^	8^d^	3				9^d^	2		1			3	14^d^		1	15
**Polygonaceae (1)**	***Rumex obtusifolius *** **L. (21)**		1	3				1	3						4			4
	Radix (4)																	
	Folium (16)	18	8					9			6	7		4	21	3	2	26
	Herba cum radice (1)			2					2						1		1	2
**Urticaceae (1)**	***Urtica dioica *** **L. (21)**		1	28				2	8	9				10	17	1	11	29
	Herba (21)																	
**Malvaceae (3)**	All Malvaceae (19)	1	23	4		1		23		5		1			26	1	2	29
	***Malva neglecta *** **Wallr. (13)**	1	21					21				1			19	1	2	22
	Herba (12)																	
	Flos (1)		1					1							1			1
	***Tilia cordata *** **Mill. (5)**			4		1				5					5			5
	Cortex (5)																	
	Others^2^ (1)		1					1							1			1
**Rubiaceae (1)**	***Coffea arabica *** **L. (16)**			18					17	1					16		2	18
	Semen (16)																	
**Boraginaceae (1)**	***Symphytum officinale *** **L. (15)**	10^e^	4					4			1	9^e^			9^e^	5		14
	Radix (13)																	
	Folium (2)	3										3			1	1	1	3
**Rosaceae (7)**	All Rosaceae (15)			10			7	7	5	2				3	16		1	17
	***Crataegus laevigata *** **(Poir.) DC. (4)**						4	4							4			4
	Herba (4)																	
	***Potentilla erecta *** **(L.) Raeusch. (4)**			5^f^					4^f^					1	5^f^			5
	Rhizoma (4)																	
	***Prunus spinosa *** **L. (3)**						3	3							3			3
	Herba (3)																	
	**Others**^**3 **^**(4)**			5					1	2				2	4		1	5
**Pinaceae (2)**	All Pinaceae (13)	5	14	7				15			3		6	2	25	1		26
	**Picea abies (L.) H. Karst. (12)**			5									4	1	5			5
	Herba (5)																	
	Resina (7)	5	14	1				15			3		2		19	1		20
	Others^4^ (1)			1										1	1			1
**Hypericaceae (1)**	***Hypericum perforatum *** **L. (12)**	7^g^	12^g^			2		14^g^		2	1	4^g^			17^g^	4		21
	Flos (12)																	
**Linaceae (1)**	***Linum usitatissimum *** **L. (11)**	1		12		1			10	2		1		1	14			14
	Semen (11)																	
**Lamiaceae (4)**	All Lamiaceae(9)	3	1	5	1		2	2	3		2	1	2	2	10	1	1	12
	***Mentha canadensis *** **L. (3)**	3^h^		2					2		2^h^	1^h^			5^h^			5
	Folium (3)																	
	***Salvia officinalis *** **L. (3)**			3				1	1				1		2		1	3
	Folium (3)																	
	Others^5^ (3)		1		1^i^		2	1					1^i^	2	3^i^	1		4
**Apiaceae (4)**	All Apiaceae (8)		2	5		1		2	4	2					8			8
	***Carum carvi *** **L. (3)**			3					3						3			3
	Fructus (3)																	
	***Sanicula europaea *** **L. (3)**		2			1		2		1					3			3
	Herba (3)																	
	Others^6^ (2)			2					1	1					2			2
**Brassicaceae (3)**	All Brassicaceae (8)	3	4	3				4	2			2		2	9	1		10
	***Brassica oleracea *** **L. (4)**	2	3					3				2			4	1		5
	Folium (4)																	
	***Brassica napus *** **L. (3)**	1	1	2				1	2					1	4			4
	Oleum (3)																	
	Others^7^ (1)			1										1	1			1
**Geraniaceae(2)**	All Geraniaceae (8)		6	4				6		2			1	1	8	1	1	10
	***Geranium robertianum *** **L.s.str. (7)**		6	3				6		2				1	7	1	1	9
	Herba (7)																	
	Others^8^ (1)			1^k^									1^k^		1^k^			1
**Amaryllidaceae (2)**	All Liliaceae (6)	1		4	1								1	5	4		2	6
	***Allium sativum *** **L. (4)**	1		3										4	2		2	4
	Bulbus (4)																	
	**Others**^**9 **^**(2)**			1	1								1	1	2			2
**Theaceae (1)**	***Camellia sinensis *** **(L.)** **Kuntze (6)**			7					7						6		1	7
	Folium (6)																	
**Adoxaceae (1)**	***Sambucus nigra *** **L. (5)**			5					4	1					5			5
	Herba (5)																	
**Fagaceae (1)**	***Quercus robur *** **L. (5)**		1	3		1		1	3	1					5			5
	Cortex (5)																	
**Aquifoliaceae (1)**	***Ilex aquifolium *** **L. (4)**						3	2						1	3			3
	Herba (3)																	
	Folium (1)			2									2		2			2
**Poaceae (1)**	***Avena sativa *** **L.s.str. (3)**	1		2					1				1	1	3			3
	Fructus (3)																	
**Rhamnaceae (1)**	***Rhamnus cathartica *** **L. (3)**						3	3							3			3
	Herba (3)																	
**Others**^**10 **^**(26)**	**26 other plant species (34)**	5	10^l^	18			6^l^	8^l^	9	3	4	3	1	11^l^	34^l^	3^l^	2	39
**Total (76)**		**71**	**164**	**159**	**2**	**11**	**21**	**182**	**94**	**36**	**18**	**37**	**14**	**47**	**359**	**37**	**32**	**428**

**I** – intact skin; **A** – alterated or sore skin; **OR** – oral; **IH** – inhalation; **IU** – intravaginal/intrauterine; **Skin** – skin afflictions and sores; **Gast** – gastrointestinal disorders and metabolic dysfunctions; **Infe** – Infertility and diseases of female genitals; **Mast** – Mastitis; **Musc** – Musculoskeletal system (including hematomas in the connective tissue; **Resp** – diseases of the respiratory tract; **No spec.** – no specification of the animal species (external administration).

^1^*Arnica chamissonis* Less. (2), *Solidago virgaurea* L. (2), *Achillea millefolium* L. (1), *Helianthus annuus* L. (1), *Tanacetum vulgare* L. (1), *Tanacetum parthenium* (L.) Sch. Bip.(1); ^2^*Althaea officinalis* L. (1); ^3^*Alchemilla vulgaris* L.Agg. (1), *Rubus idaeus* L. (1), *Malus domestica* Borkh.(1), *Prunus domestica* L. (1); ^4^*Abies alba* Mill. (1); ^5^*Lavandula angustifolia* Mill. (2), *Thymus vulgaris* L. (1); ^6^*Petroselinum crispum* (Mill.) Fuss (1), *Foeniculum vulgare* Mill. (1); ^7^*Capsella bursa- pastoris* (L.) Medik (1); ^8^*Pelargonium sidoides* DC (1); ^9^*Allium cepa* L. (2); ^10^*Beta vulgaris subsp. vulgaris* Conditiva group (1) (Amaranthaceae); *Dryopteris filix-mas* (L.) Schott (1) (Aspidiaceae); *Betula pendula* Roth (1) (Betulaceae); *Cannabis sativa* L. (1) (Cannabaceae); *Chenopodium bonuscphenricus* L. (1) (Chenopodiaceae); *Juniperus communis* L.s.str. (2) (Cupressaceae); *Thuja occidentalis* L. (2) (Cupressaceae); *Equisetum arvense* L. (1) (Equisetaceae); *Vaccinium myrtillus* L. (2) (Ericaceae); *Anthyllis vulneraria* L.s.str. (1) (Fabaceae); *Gentiana lutea* L. (1) (Gentianaceae); *Juglans regia* L. (1) (Juglandaceae); *Cinnamomum verum* J.Presl (1) (Lauraceae); *Lycopodium clavatum* L. (1) (Lycopodiaceae); *Myristica fragrans* Houtt. (2) (Myristicaceae); *Melaleuca alternifolia* Maiden&Betche ex Cheel (2) (Myrtaceae); *Fraxinus excelsior* L. (2), *Olea europaea* L. (1) (Oleaceae); *Pedicularis verticillata* L. (1) (Orobanchaceae); *Euphrasia officinalis* L. (1), *Plantago lanceolata* L. (2) (Plantaginaceae); *Citrus x limon* (L.) Burm.f. (1) (Rutaceae); *Salix caprea* L. (1) (Salicaceae); *Quassia amara* L. (1) (Simaroubaceae); *Solanum tuberosum* L. (2) (Solanaceae); *Tropaeolum majus* L. (1) (Tropaeolaceae);^ 11^parasites, general strengthening, behaviour, sensory organs, varia; ^12^horses, pigs, donkeys, goats, sheep, dogs, hens, rabbits.

^a^Kamillosan® Liquidum used for two applications; ^b^Calendula tincture (pharmacy) used for two applications, calendula unguent (pharmacy) used for five applications; ^c^Arnica oil (pharmacy) used for one application, arnica ointment (pharmacy) used for one application; ^d^Goldenrod unguent (pharmacy) used for three applications; ^e^Comfrey emulsion (pharmacy) used for one application; ^f^Common tormentil tincture (pharmacy) used for one application, ^g^St John's wort oil (pharmacy) used for four applications; ^h^NPJ Liniment® and OPIFIX® (both containing Mentha arvensis L. var. piperascens) used for three applications; ^1^Thyme oil used for one application; ^k^Pelargonium Spray® used for one application;^1^Tea Tree oil used for two applications.

*Calendula officinalis* L. (42 applications), *Malva neglecta* Wallr. (21 applications), *Matricaria recutita* L. (15 applications), *Picea abies* (L.) H. Karst. (resin; 15 applications), and *Hypericum perforatum* L. (14 applications) were the most frequently used plants for treatment of skin afflictions and sores. The highest number of uses for treatment of gastrointestinal disorders and metabolic dysfunctions was listed for *Coffea arabica* L*.* (17 applications, always in conjunction with schnaps), *Matricaria recutita* L*.* (13 applications) and *Linum usitatissimum* L. (10 applications). For treatment of injuries of the musculoskeletal system (incl. hematoma of connective tissue) *Symphytum officinale* L. (12 applications), *Rumex obtusifolius* L. (7 applications) and *Arnica montana* L. (5 applications) were most often mentioned. *Urtica dioica* L. (9 applications), *Matricaria recutita* L. (5 applications) and *Tilia cordata* Mill. (5 applications) were most frequently mentioned for treatment of infertility and diseases of female genitals (Table 3).

Ointments containing *Rumex obtusifolus* L. (6 applications) and *Picea abies* (L.) H. Karst. (3 applications) were most frequently mentioned in the treatment of mastitis, and *Picea abies* (L.) H. Karst. (6 applications) was most often mentioned for treatment of respiratory tract diseases (Table 3).

Out of 428 applications 359 were for treatment of cattle (83.9%), and 32 applications (7.5%) were used for other animal species. No specific animal species were mentioned for 37 applications (8.6%) all for the treatment of skin afflications and sores (Table 3).

### Route of administration of the 428 applications of the 315 HSHR

More than half of all administrations were external, mainly on altered and sore skin including claws, hooves, navels and conjunctiva (164 applications, 38.3%). For these treatments the farmers used the preparations as a bath, compress, wash, or simply as a direct application of the fresh plants or oils, ointments and tinctures thereof. Administration on intact skin was reported in 71 applications (16.6%), mainly to treat internal injuries like pulled muscles, contusions, sprains, swellings and tensions, mastitis or as repellent against ectoparasites. Some farmers reported that they rubbed the calves’ small of the back with an oil or ointment to treat inflammations of the navel (Table 3).

Oral administration of HSHR was reported for 159 applications (37.1%), mainly to treat diarrhoea, stomach trouble, indigestions, flatulence, cough, infertility, and diseases of female genitals (including the cleaning of the uterus after calving), or for general strengthening. Orally HSHR were either added to the feedstuff or constrained oral applicated. A total of 11 applications (2.6%) were intravaginal/ intrauterine, to prevent or treat an inflammation of the uterus, or for cleaning the uterus after calving. Two preparations (0.5%) were used for inhalative purposes to treat ailments of the respiratory tract (Table 3).

A total of 21 preparations (4.9%) were used in the stable and surrounding area, without direct contact to the animal itself: To combat cattle ringworm, twigs of *Crataegus laevigata* (Poir.) DC., *Prunus spinosa* L., *Rhamnus cathartica* L. or *Ilex aquifolium* L. were suspended in the stable for several weeks. The farmers used these both as a prophylactic and therapeutic measure. Other treatments of housing environments were used as measures to prevent flu, or as repellent for parasites (Table 3).

### Further information regarding applications

For all applications, the date of their last use was queried. More than 60% of the applications have been used within the last year preceding the interviews. More than a year ago, but within the last ten years, additional 114 applications (26.6%) had been used. In 53 cases (12.4%) the last applications was more than ten years ago or only heared of by the dialog partners. Additionally, we enquired about the frequency of use within the last five years. About one third of all preparations (160; 37.4%) had been used by the farmers more than ten times, and 69 applications (16.1%) between six and nine times during the last five years. A total of 77 (18.0%) had been used between two and five times, and 122 applications (28.5%) had been employed less than two times.

In 272 applications (63.5%) the farmers used the HSHR without other accompanying therapies. A total of 156 applications (36.5%) were used in combination with other herbal remedies or homeopathic preparations: three quarters of these HSHR were always used in combination, whereas for the remaining cases a combination with other preparations depended on the specific condition of the animal to be treated. In wound care the combination of two HSHR was common. A typical treatment consisted of cleaning of the wound with one HSHR, and subsequent application of a second HSHR, such as an ointment. If the condition of the animal further deteriorated during treatment, the farmers called a veterinarian.

In 48.6% of cases (208 applications) the knowledge on the use of the applications was obtained from ancestors and relatives. Information obtained from friends accounted for additional 77 uses (18.0%). In 58 cases (13.6%) knowledge was acquired through own practical experience. In other cases knowledge was obtained from attending courses (51 applications, 11.9%), from books and journals (13 applications, 3.0%), and others sources (20 applications, 4.7%).

**Figure 2:**
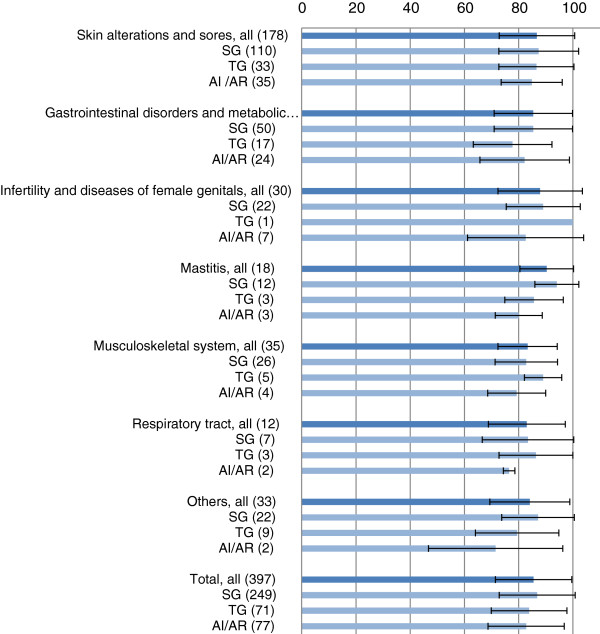
**Degree of satisfaction of users with their treatment outcome based on a Visual Analogue Scale (mm VAS), mean value and the standard deviation of the VAS are represented.** SG = St. Gallen, TG = Thurgau, AI/AR = Appenzell Innerrhoden and Appenzell Ausserrhoden.

The degree of satisfaction of users with the outcome of their treatments could be recorded for a total of 397 applications (Figure 2). In 31 cases it was not possible to assess the degree of satisfaction, e.g. if the last use of a remedy had been long time ago. An average VAS value of over 80 mm represents a high degree of satisfaction.

## Discussion

We collected here for the first time detailed information regarding the knowledge and use of homemade herbal remedies in four cantons located in north-eastern Switzerland (St. Gallen, Thurgau, Appenzell Innerrhoden and Appenzell Ausserrhoden).

The methodology used in an earlier survey conducted in a different part of Switzerland [16] was also applied in this study and, again, proved to be suitable. A focus on organic farms was appropriate, since “phytotherapeutic […] products” are one of the preferred methods for the treatment of livestock diseases [21], but on the opposite only very few herbal medicinal products are approved for veterinary purposes [23]. For these reasons organic farmers may be more motivated to prepare their own herbal remedies than non-organic. Thanks to public presentations of the project and to snowball sampling [30] non-organic farmers could also be recruited as interview partners. Interviews were conducted on 17 out of a total of 760 (2.2%) organic farms, and on 21 out of 8127 (0.3%) non-organic farms in the research area. As expected, organic farms were overrepresented in the study, due the approaches used for recruitment of interview partners. The percentage of organic farms participating in this study was comparable with that in an earlier survey [16].

The snowball sampling method is commonly used in ethnoveterinary research [12-17,40]. However, the farms included may not be representative for all farms of the region to be studied.

Interviews were conducted in March and April, in order to avoid farmers’ work peaks during summer. A disadvantage of the timing was that plant material was not available, except when farmers kept dried plant material. However some voucher specimen could be generated during the following summer.

According to the research in Aargau, Zurich and Schaffhausen [16] this survey determines the dosage and concentration of the used plant, which are not documented elsewhere. This enables to make a comparison with literature regarding recommended dose and concentration respectively (Tables 4 and 5). There was a wide range of dosages and concentrations reported. The high therapeutic index of most herbal drugs may be one explanation. The therapeutic index describes the span of dosage between first therapeutic effects and first toxic effects. As larger the span is as less is the risk compared to the potential therapeutic profit.

**Table 4:** **Daily dosage in dry plant equivalent per kg metabolic body weight (g/kg**^**0.75**^**) of homemade single species herbal remedies (HSHR) used in orally administered preparations**

**Plant species with ≥ 3 reported HSHR and documented dosage**	**Daily dose [g/kg**^**0.75**^**]**				**Determined daily dose [g/kg**^**0.75**^**] in Aargau, Zurich and Schaffhausen (arithmetic mean) **[16]	**Converted animal daily dose [g/kg**^**0.75**^**] (Reichling et al. **[41]**)**	**Converted human daily dose [g/kg**^**0.75**^**] (ESCOP **[42]**)**
	**Calf (75 kg)**	**Cattle (650 kg)**	**Others**^**1**^	**Arithmetic mean (median; minimum value- maximum value)**			
	**(MWB = 25.5 kg**^**0.75**^**)**	**(MBW = 128.7 kg**^**0.75**^**)**					
***Coffea arabica *L.** Semen (18)	0.04	0.04, 0.06, 0.06, 0.07,	0.11^2^,1.58^3^	**0.35**	0.37	-	-
		0.09, 0.13, 0.19, 0.19,					
		0.31, 0.31, 0.45, 0.45,		(0.19; 0.04-1.58)			
		0.51, 0.71, 1.06					
***Urtica dioica *L.** Herba (18)	0.55, 2.21	0.02, 0.02, 0.03, 0.10,	0.06^4^, 0.53^5^, 0.79^6^, 0.90^5^, 1.05^4^	**0.49**	2.39	0.19-0.39^7^	0.35-0.52
		0.16, 0.22, 0.22, 0.26,		(0.26; 0.02-2.21)		0.39-0.98^8^	
						0.19-0.38^4^	
		0.26, 0.43, 0.96					
***Matricaria recutita *L.** Flos(13)	0.12, 0.16,	0.17, 1.94		**1.12**	0.22	0.19-0.39	0.39-0.52
	0.16, 0.35,						
	0.47, 0.53, 0.68,						
				(0.53; 0.12-5.88)			
	0.79,1.32,						
	2.00, 5.88						
***Linum usitatissimum *L.** Semen (12)	4.41, 4.41,	0.62, 0.78, 1.57, 1.62,		**5.16**	2.92	0.39-1.55^7^	0.66
	5.12, 10.97,					0.98-1.96^8^	
		1.62, 7.78, 7.78		(4.21; 0.62-15.69)			
	15.69						
***Camellia sinensis *(L.)Kuntze** Folium (7)	0.12, 0.53, 0.56,		0.18^9^	**0.64**	-	1.67-6.67^11, 12^	-
	0.62, 0.71, 1.74			(0.56; 0.12-1.74)			
***Potentilla erecta *(L.) Raeusch.** Rhizoma (4)	0.004, 0.004	0.11, 0.44		**0.14**	-	0.16-0.31^7^	-
				(0.06; 0.004-0.44)		0.20-0.59^8^	
***Allium sativum *L.** Bulbus (3)	1.76		0.10^10^, 1.50^10^	**1.12**	-	0.16-0.23	0.09-0.17
				(1.50; 0.10-1.76)			
***Carum carvi *L.** Fructus (3)	3.15	0.11, 0.14		**1.13**	1.13	0.19-0.39	0.07-0.26
				(0.14; 0.11-3.15)			
***Geranium robertianum *L.s.str.** Herba (3)	0.001	0.02, 0.11		**0.04**	-	-	-
				(0.02; 0.001-0.11)			
***Rumex obtusifolius *L.** Radix (3)	0.82, 1.18, 3.15			**1.72**	-	-	-
				(1.18; 0.82-3.15)			

^1^hens, pigs, dogs, rabbit, sheeps, goats and donkeys, ^2^young sheep, ^3^rabbits, ^4^pigs, ^5^goats, ^6^donkeys, ^7^cattle, ^8^calves, ^9^dogs, ^10^hens, ^11^rats, ^12^Besra et al.: Antidiarrhoeal Activity of Hot Water Extract of Black Tea (*Camellia sinensis*) [50].

**Table 5:** Concentration of medicinal plants in homemade single species herbal remedies (HSHR) in preparations for topical use

**Plant species with ≥ 3 HSHR and documented dosage**	**g dry plant equivalent in 100 g finished product**				**Recommended concentration g dry plant equivalent in 100 g finished product **[41]**,**[42]**,**[48]**,**[49]		
	**Extraction** **with water**	**Extraction with alcohol**	**Extraction with oil/fat**	**Arithmetic mean (median; minimum value - maximum value)**	**Extraction with water**	**Extraction with alcohol**	**Extraction with oil/fat**
***Calendula officinalis *L.** Flos and flos sine calice (21)	0.10, 0.20	0.01, 0.03, 0.34, 0.38, 0.43, 0.91, 2.33	0.28, 0.34, 0.50, 1.22,	**1.10**	0.67-1.33 [42]	50.00^1^[42]	1.00-5.00^3^[42]
			1.52, 1.73, 1.76, 1.82,				
						20.00^2^[42]	
				(0.91; 0.01-3.27)			
			1.82, 1.87, 2.22, 3.27				
***Rumex obtusifolius *L.** Folium (13)			1.52, 1.52,	**4.31**	-	-	-
			1.75, 1.82,				
			1.98, 2.00,				
			2.33, 2.77,	(2.33; 1.52-20.00)			
			3.08, 3.59,				
			3.64, 10.00, 20.00				
***Hypericum perforatum *** **L.**			0.62, 0.66,	**1.58**	5.00- 10.00^4^[41]	-	5.00^5^[49] 11.00^4^[48]
			0.76, 1.43,				
				(1.69; 0.62-3.00)			
			1.49, 1.89,				
Flos (10)							
			1.96, 2.00,				
			2.00, 3.00				
***Matricaria recutita *L.** Flos (10)	0.04, 0.18, 0.23, 0.37, 0.40, 0.40, 0.85, 5.00		2.12, 5.65	**1.52**	0.5 [42]	-	-
				(0.40; 0.04- 5.65)			
***Symphytum officinale *L.***Radix* (8)		3.51, 4.26, 4.43, 8.33	1.82, 3.45, 6.35, 6.67	**4.85**	-	-	up to 17.5 [42]
				(4.35; 1.82-8.33)			
***Malva neglecta *Wallr.** Herba (6)	0.20, 0.40, 0.40, 0.80		1.82, 1.82	**0.79**	-	-	-
				(0.40; 0.10-1.82)			
Flos (1)	0.10						
***Arnica montana *L.** Flos (6)		0.90, 0.91, 1.14, 1.96, 2.33	10.00	**2.87**	2.0 [42]	10.00-33.33 [42]	-
				(1.55; 0.90-10.00)			
***Geranium robertianum *L.s.str.** Herba (4)			0.71, 0.74, 0.90, 0.94	**0.82**	-	-	-
				(0.82; 0.71-0.94)			
***Picea abies *(L.) H. Karst.** Resina (4)			3.53, 12.93, 27.27, 31.75	**18.87**	-	-	-
				(20.10; 3.53- 31.75)			

^1^40% ethanol, ^2^90% ethanol, ^3^semi-solid preparations, ^4^herb, ^5^flowering twigs.

About 49% of the applications are based on the knowledge of ancestors and relatives which represents a transmission over at least two generations. To document the transmission over more than these both generations as proposed by some authors [19] assumes that the dialogue partner at the moment of the interview still knows, the origin of the knowledge of his ancestor which will be not often the case. The use of a visual analogue scale (VAS) to estimate satisfaction of farmers with the outcome of their treatments certainly gave only subjective results, and not an objective assessment of therapeutic success. However, the generally high degree of satisfaction became obvious, and was in accord with observations in the earlier study [16].

*Matricaria recutita* L. and *Calendula officinalis* L. were the most frequently used plants. This finding was in accord with earlier surveys conducted in Switzerland and Austria [13-17]. Likewise, *Coffea arabica* L*., Hypericum perforatum* L., *Linum usitatissimum* L*., Symphytum officinale* L. and *Urtica dioica* L. were among the frequently used plants in these surveys [13-17]. In contrast, use of *Arnica montana* L. was more often reported in our study than in the previous survey conducted in Aargau, Zurich and Schaffhausen [16]. Arnica grows naturally in the alpine regions and is likely better known and accessible to local farmers. An even higher number of uses of arnica was reported from Safiental and Austria [14,15,17]. *Rumex obtusifolius* L., *Geranium robertianum* L.s.str. and *Camellia sinensis* (L.) Kuntze were often mentioned in our survey, but were much less used in central-northern Switzerland [16]. *Malva neglecta* L., and resin from *Picea abies* (L.) H. Karst. were mentioned several times by farmers, and were also frequently used by farmers of Safiental [17]. In contrast, remedies based on plants of the *Apiaceae* and *Lamiaceae* families were less frequently reported in the present study than in the survey carried out in central-northern Switzerland [16].

The order of the most frequently mentioned categories of use was the same in our survey as in the previous study. However, the percentage of administration on altered and sore skin was more often reported, and oral administrations were less frequently documented in our survey [16].

The ten most often mentioned medicinal plants are discussed in the following sections. The dosage in case of oral administrated applications, respectively the concentrations of topical administrated applications as well as the categories of use are focused.

### Chamomile flowers (*Matricaria recutita* L., Matricariae flos)

Chamomile was administered internally and externally to treat gastrointestinal diseases, skin afflictions and sores, and infertility and diseases of female genitals. Chamomile was previously documented in ethnoveterinary surveys from Switzerland, Austria, southern Italy, and western Spain [2,8,9,12-17]. In the majority of cases it was used to treat gastrointestinal diseases and skin afflictions, but also the treatment of infertility and diseases of female genitals have been reported [13,15]. Ethnoveterinary use of chamomile was not documented in surveys conducted in Catalonia, Andalusia, Galicia and Tuscany [5-7,11]. *Chamaemelum nobile* L., which can be considered as the Mediterranean equivalent of *Matricaria recutita* L., has been used in Navarra, eastern Spain as an orally administered infusion to treat bloating of ruminants in spring and other diseases [4].

In veterinary medicine the use of chamomile has been reported in the treatment of gastrointestinal disorders, metabolic dysfunction, and skin afflictions and sores [41]. These uses are supported by *in vitro* and *in vivo* pharmacological studies [42,43]. In our survey, daily dosages for oral administration reported by farmers were in average higher than the recommended veterinary daily doses [41] and the human daily doses [42]. In addition, the daily dosages were also higher than the mentioned daily doses in the previous survey in central-northern Switzerland [16] (Table 4). The concentrations used in formulations for topical treatment were, in average, higher than the recommended concentration [42] (Table 5).

### Marigold flowers (*Calendula officinalis* L., Calendulae flos)

The farmers prepared tinctures, oils, ointments and infusions from marigold flowers, and these preparations were mostly used to treat skin afflictions and sores. These uses correspond with those documented in surveys conducted in other parts of Switzerland and in Austria [13,14,16,17].

Use of marigold preparations was mentioned at least once in the treatment of gastrointestinal disorders, diseases of female genitals, and injuries of the musculoskeletal system. Treatment of gastrointestinal disorders, and injuries of the musculoskeletal system has also been documented in Austria [13]. Use in the treatment of wounds has been described in veterinary medicine [41]. These uses are supported by findings from *in vitro* and *in vivo* pharmacological studies [42,44,45].

In topical treatment with lipophilic products (extraction with oil or fat) the dosage of herbal drug was within the range recommended by the literature [42]. Within contrast, the concentration of marigold preparations obtained by acqueous or alcoholic extraction were lower than recommended by the ESCOP monograph [42] (Table 5).

### Broad-leaved dock (*Rumex obtusifolius* L.)

Decoctions prepared from roots of broad-leaved dock were orally administered for treatment of gastrointestinal disorders, and externally for skin afflictions and sores. Gastrointestinal disorders were also treated by administration of the entire plant with attached roots. Leaves were used, either by direct application onto skin, or after processing to an ointment, to treat skin afflictions and sores, mastitis, and injuries of the musculoskeletal system. The interview partners highlighted the cooling effect of the leaves, which they considered particularly favourable in case of inflammations.

Earlier surveys conducted in Austria and other areas of Switzerland reported the use of broad-leaved dock as a treatment for diarrhoea, or as ointments for injuries [12,14,16,17]. Use of other *Rumex* species has been documented in Catalonia, Tuscany and Austria [7,11,12].

The calculated average daily dose for oral application of dry plant equivalent was 1.7 g/kg^0.75^ (Metabolic Body Weight; MBW), and the concentration in ointments for topical use was 4.3 g dry plant equivalent/100 g finished product (Tables 4 and 5). No dose recommendations for broad-leaved dock could be found in monographs or in scientific literature.

### Stinging nettle (*Urtica dioica* L.)

The herb of stinging nettle was orally administered either directly, or as an infusion. Uses were reported in cattle, goats, pigs and donkeys. It was thus the only herbal drug used to treat four different animal species. This is in line with the findings of an earlier study [16]. Stinging nettle herb was used in cases of infertility, diseases of female genitals, gastrointestinal disorders, metabolic dysfunction, and for general strengthening. In one case stinging nettle was applied externally for treatment of altered or sore skin. Stinging nettle is widely used in Europe, since ethnoveterinary reports documented this herb also in other parts of Switzerland, in Spain, Italy, and Austria [2,4-7,11,13-17]. Veterinary medicine recommends the internal use of stinging nettle herb to increase urinary flow during bacterial and inflammatory diseases, and as an orally or externally administered adjuvant treatment in rheumatic ailments. Stinging nettle herb reportedly shows antihypertensive, analgesic, local anesthetic, antiphlogistic, antirheumatic and diuretic properties [41,42].

The average oral dosages reported by our interview partners were slightly higher for cattle and pigs than the recommended dose, and were comparable to recommended veterinary doses for calves [41,42] (Table 4). Compared to the daily doses documented in Aargau, Zurich and Schaffhausen (2.4 g/kg^0.75^ (MBW)) [16], lower average doses were used by farmers in our survey (0.5 g/kg^0.75^ (MBW)) (Table 4).

### Coffee beans (*Coffea arabica* L.)

An infusion of toasted coffee beans (coffee) was always administered orally and in combination with schnaps to treat gastrointestinal disorders, metabolic dysfunction, infertility, and diseases of female genitals. In other studies in Switzerland and Austria coffee with, but also without schnaps, has been documented for similar traditional uses [13-17].

The daily dosage of dry plant with an arithmetic mean of 0.4 g/kg^0.75^ (MBW) is in a comparable range with the daily dosage found in a previous survey conducted in Switzerland [16] (Table 4).

### Comfrey (*Symphytum officinale* L.)

Preparations made from comfrey root were externally applied in case of injuries of the musculoskeletal system, skin afflictions, and sores and mastitis. Roots were either used freshly crushed, or as extracts prepared with alcohol, oil or fat. Leaves were applied directly onto skin to treat injuries of the musculoskeletal system.

Comparable uses have been previously reported from Switzerland and Austria [13-17]. Phytoveterinarian literature recommends topical use of comfrey preparations for treatment of contusions, sprains, and pulled muscles [41,42]. The documented concentrations in g dry plant equivalent per 100 g finished product were lower than the recommended concentration in literature [42] (Table 5).

### Common Mallow (*Malva neglecta* Wallr.)

Common mallow was mostly used as an infusion, but maceration in water, oil or fat were occasionally also mentioned. It was mainly applied for treatment of altered or sore skin, in particular abscesses of claws and, in one case, for treatment of injuries of the musculoskeletal system.

No formulations had been documented from a survey in central-northern Switzerland [16], but treatment of abscesses and wounds had been reported from Safiental, Austria, and northern Spain [4,13,15,17]. In Austria common mallow infusions were also administered orally to treat gastrointestinal disorders [15].

A mean concentration of 0.8 g dry plant equivalent/100 g finished product was calculated. No recommended concentration in literature was found (Table 5).

### St. John's wort (*Hypericum perforatum* L.)

Farmers prepared oils and ointments with St. John’s wort. The interview partners used only flowers, and this was in accord with reports from Safiental and Austria [15,17]. Preparations were used for treatment of skin afflictions and sores, and these uses had also been reported from others parts of Switzerland, Austria, Italy and Spain [4,6,7,9,11,13-17]. Treatments of diseases of female genitals, mastitis, and injuries at the musculoskeletal system were also mentioned by our interview partners, albeit less frequently. Treatment of mastitis and injuries at the musculoskeletal system was previously documented from other parts of Switzerland and from Austria [13,16,17].

St. John’s wort shows antidepressant, antibacterial, antiviral, antiproliferative, and anti-inflammatory properties, and the oil is recommended to treat wounds [41]. The wound- healing activity is supported by in vivo pharmacological studies [46,47]. St. John’s wort contains naphthodianthrons, flavonoids and tannins [41].

In literature mainly preparations of the flowering herb and not only of the pure flowers are documented [41]. The flowers of *Hypericum perforatum* L. contain a higher percentage of the component Hyperforin, which shows antibiotic activities than the herb [48] or probably also than the blossoming St John’s wort tips [49]. This could explain the difference in the concentration compared with the literature (Table 5).

### Common spruce (*Picea abies* (L.) H. Karst.)

Twigs of common spruce were directly fed to calves to treat or prevent cough and pneumonia.

Ointments prepared from resin were used to treat skin afflictions and sores, mastitis, and respiratory tract diseases. To treat cough or pneumonia in calves, farmers rubbed ointment onto the chest of sick animals. Use of twigs of common spruce for treatment of respiratory tract diseases, and of resin to treat wounds has been presviously reported from other parts of Switzerland, and from Austria [13,14,16,17].

A mean concentration of 18.9 g dry plant equivalent/100 g finished product was documented (Table 5). No literature of recommended concentration of the resin was found.

### Linseeds (*Linum usitatissimum* L.)

Linseeds were either used directly, or as infusions and decoctions. They were administered orally against gastrointestinal disorders, infertility, diseases of female genitals, and for general strengthening. Intravaginal/intrauterine administration was mentioned to treat inflammation of the uterus. Externally applied infusions and decoctions were used to treat injuries of the musculoskeletal system. Similar uses of linseed have been reported from Safiental, Austria, and Tuscany [11,13-15,17].

Linseeds contain mucilaginous polysaccharides which produce a protective and soothing layer on skin and mucous membranes. In veterinary medicine, linseeds are used as a mild laxative [41].

The dosages administered by the farmers interviewed in our study were higher than those reported in other surveys, or recommended daily dosages in veterinary medicine [16,41,42] (Table 4).

## Conclusions

Farmers in north-eastern Switzerland possess traditional knowledge on medicinal plants and their uses in the treatment of livestock. We documented in our survey a wide spectrum of plant species, preparations, and uses. A considerable part of the documented remedies and their applications is in accordance with established pharmacological effects. Compared to ethnoveterinary studies previously conducted in other parts of Switzerland, and in Austria, similar plants and uses were found, but also additional plants and uses could be documented. The interviewed farmers were mostly satisfied with the outcome of their applications.

A continued documentation of traditional knowledge in other parts of Switzerland and Europe is needed.

## Abbreviation

HSHR: Homemade single species herbal remedies.

## Competing interests

The authors declare that they have no competing interests.

## Authors' contributions

MD conducted all interviews of the project, entered the results into the database, performed the data analysis, and drafted the manuscript. Together with CV and BM, MW and FK designed the research strategy. SI created and took care of the project database. AT collected, processed and labeled the voucher specimens. MH, MW, CV, SI, FK and BM critically revised the manuscript for scientific content, and MH did the final language check. All authors read and approved the final manuscript.

## Supplementary Material

Additional file 1 **List of remedies and their applications.** Ethnoveterinary herbal remedies used by farmers in four north-eastern Swiss cantons (St. Gallen, Thurgau, Appenzell Innerrhoden and Appenzell Ausserrhoden). Click here for file
