# Supplementary material for: Ethnoveterinary herbal remedies used by farmers in four north-eastern Swiss cantons (St. Gallen, Thurgau, Appenzell Innerrhoden and Appenzell Ausserrhoden)
Source: J Ethnobiol Ethnomed. 2014 Mar 31;10:32. doi: 10.1186/1746-4269-10-32 (PMC4022237; doi:10.1186/1746-4269-10-32)
Supplement: Additional file 1 — List of remedies and their applications. Ethnoveterinary herbal remedies used by farmers in four north-eastern Swiss cantons (St. Gallen, Thurgau, Appenzell Innerrhoden and Appenzell Ausserrhoden). [file 1746-4269-10-32-S1.pdf]

List of remedies and their applications

Ethnoveterinary herbal remedies used by farmers in four north-eastern Swiss cantons (St. Gallen, Thurgau, Appenzell Innerrhoden and Appenzell Ausserrhoden)

One line in the following table represents one application: [dialogue partner] x [plant species] x [plant part] x [manufacturing process to the finished product] x [category of use] x [specification of use] x [animal species] x [animal age classification] x [administration procedure]. The [specification of use] is not included in the additional file 1 for space reasons. This explains identical lines.

List of abbreviations

General:
na= information not available

Plant part:
bar= bark
exc= excretions
flo= flowers and inflorescenses
fsb= fruits, seeds, berries
her= whole plants without roots (herb)
lea= leaves
pet= petals
rob= root/ bulb
twb= twigs, branches
wpr= whole plants with roots

Origin:
bo= bought/ crude drug
bo*= bought/ commercial extracts and finished products
cu= cultivated
wh= wild harvesting

vs no: Number of Herbarium voucher

Extraction procedure on farm:
none
alcohol: (rt)= room temperature
(et)= elevated temperature
milk: (rt)= room temperature
(et)= elevated temperature
oil/fat: (rt)= room temperature
(et)= elevated temperature
water : (rt)=room temperature
(dec)=decoction
(inf)= infusion

Categories of use:
behav=behaviour
gastr= gastrointestinal disorders and metabolic dysfunctions
infer=infertility and diseases of female genitals
mast= mastitis
musc= musculoskeletal system
para=parasites
resp=respiratory tract diseases
sens= sensory organ
skin= skin alterations and sores
streng= general strengthening
varia= various indications

Animal treated:
nsas= no specification of the animal species (external administration)

Administration
exal= external administration, altered or sore skin
(epicutan)
(konj)= konjuntival
exin= external administration, intact skin
(epicutan)
(konj)= konjunktival
int= internal administration
(nasal)
(oral)
(vaut)= intravaginal/ intrauterine
tohe= treatment of housing environment

Daily dosage [g/kg$^{0.75}$]
in plant equivalent per kg metabolic body weight [g/kg$^{0.75}$];
used in formulations for oral administration only;
tohe= treatment of housing environment

RN= Recipe number (starts with 202)
DP= Dialog partner (starts with 30)

Conc [g/100g]
Concentration [g drug in 100g finished product]; used in formulations for topical treatment and intravaginal/ intrauterine administration;
da= directly administered without extraction, external administration
tohe= treatment of housing environment

Ver= Verification of dosage
ew= estimated weight by assessement of the volume and subsequent weighing
od= original drug weighted on- site
rd= reference drug weighted on- site

Botanical family	Plant species	Recipe name designated by DP	Plant part	Origin	Extraction procedure on farm	Categories of use	Animal treated	Administration	Daily dosage [g/kg0.75]	Conc [g/100g]	Ver	RN	DP
**Adoxaceae**	*Sambucus nigra* L.	Holderstaude	twb	wh	none	gastr	cattle	int (oral)	na		na	705	47
		Holderstaude							na		na	704	46
		Holderstaude							na		na	582	65
		Holderstaude							na		na	264	34
		Holundertee			water (inf)	infer	cattle	int (oral)	0.02		ew	426	61
**Amaranthaceae**	*Beta vulgaris subsp. vulgaris* (conditiva- group)	Randenwickel	rob	cu	none	musc	cattle	exin (epicutan)		da	na	269	35
**Amaryllidaceae**	*Allium cepa* L.	Zwiebeln	rob	cu	none	resp	calf	int (nasal)	na	na	na	281	36
		Zwiebeln				streng	cattle	int (oral)	1.63		ew	302	39
	Allium sativum L.	Knoblauch	rob	bo	milk (rt)	para	calf	int (oral)	1.76		od	548	73
		Knoblauch			none	para	hen	int (oral)	1.5		ew	318	40
		Knoblauch- Wasser			water (rt)	para	cattle, calf	exin (epicutan)		0.15	ew	610	54
		Knoblauch		cu	none	para	hen	int (oral)	0.1		ew	312	39
**Apiaceae**	*Carum carvi* L.	Kümmeltee	fsb	bo	water (dec)	gastr	calf	int (oral)	3.15		od	675	48
		Kümmeltee					cattle	int (oral)	0.14		rd	544	73
		Kümmeltee		wh	water (inf)	gastr	cattle	int (oral)	0.11		rd	429	61
	Foeniculum vulgare Mill.	Fencheltee	fsb	bo	water (inf)	gastr	calf	int (oral)	0.59		rd	321	43
	Petroselinum crispum (Mill.) Fuss	Peterli- Tee	her	cu	water (inf)	infer	cattle	int (oral)	0.03		ew	342	45
	Sanicula europaea L.	Sarnikel- Tinktur	her	wh	alcohol (rt)	skin	cattle, calf	exal (epicutan)		0.01	od	278	36
		Sanikel- Tee			water (inf)	infer	cattle	int (vaut)		na	na	209	30
		Sarnikel- Tee				skin	cattle	exal (epicutan)		0.1	od	279	36
**Aquifoliaceae**	*Ilex aquifolium* L.	Palmentee	lea	wh	water (dec)	resp	calf	int (oral)	0.01		ew	679	48
		Palmentee					cattle	int (oral)	0.01		ew	679	48
		Stechpalme	twb	wh	none	para	calf	tohe	tohe	tohe	na	282	36
		Stechlaub						tohe	tohe	tohe	na	358	46
		Stechpalme						tohe	tohe	tohe	na	454	66
**Aspidiaceae**	*Dryopteris filix-mas* L. (Schott)	Farn	her	wh	none	para	cattle, calf	tohe	tohe	tohe	na	364	51
**Asteraceae**	*Achillea millefolium* L.	Schafgarben- Tee	flo	bo	water (inf)	gastr	calf	int (oral)	0.53		od	701	47
		Schafgarben- Tee					cattle	int (oral)	0.19		od	701	47
	Arnica chamissonis Less.	Arnikatinktur	flo	cu	alcohol (rt)	skin	cattle	exal (epicutan)		0.23	ew	569	68
		Arnikasalbe			oil/fat (rt)	skin	cattle, calf	exal (epicutan)		2	ew	570	68
		Arnikasalbe								2	ew	570	68
	Arnica montana L.	Arnikabalsam	flo	bo*	none	musc	cattle, calf	exin (epicutan)		na	na	297	37
		Arnikaöl								na	na	617	54
		Arnikaschnaps		wh	alcohol (rt)	musc	cattle	exin (epicutan)		2.33	ew	542	73
		Arnikatinktur					nsas	exin (epicutan)		0.9	ew	638	52
		Arnikatinktur				skin	cattle	exal (epicutan)		1.14	ew	391	57
		Arnikatinktur								1.14	ew	391	57
		Arnikaschnaps					cattle, calf	exal (epicutan)		0.91	ew	655	51
		Arnikatinktur					nsas	exal (epicutan)		1.96	ew	341	45
		Arnikatinktur								1.96	ew	341	45
		Arnikasalbe			oil/fat (rt)	musc	nsas	exin (epicutan)		10	od	639	52

Botanical family	Plant species	Recipe name designated by DP	Plant part	Origin	Extraction procedure on farm	Categories of use	Animal treated	Administration	Daily dosage [g/kg0.75]	Conc [g/100g]	Ver	RN	DP
**Asteraceae**	*Calendula officinalis* L.	Ringelblumensalbe	flo	bo*	none	skin	calf	exin (epicutan)		na	na	597	58
		Calendulasalbe					cattle	exal (epicutan)		na	na	690	46
		Calendulasalbe								na	na	690	46
		Calendulatinktur								na	na	691	46
		Calendulatinktur								na	na	691	46
		Ringelblumensalbe								na	na	410	60
		Ringelblumensalbe					nsas	exal (epicutan)		na	na	410	60
		Ringelblumentinktur		cu	alcohol (rt)	infer	cattle	int (vaut)		2.5	ew	367	54
		Calendulatinktur				skin	calf	exal (epicutan)		0.38	ew	672	48
		Ringelblumentinktur								0.34	ew	219	32
		Ringelblumenschnaps					cattle	exal (epicutan)		0.91	ew	654	51
		Ringelblumentinktur								0.03	ew	277	36
		Ringelblumentinktur								0.34	ew	219	32
		Ringelblumentinktur								0.43	ew	496	72
		Calendulatinktur					cattle, calf	exal (epicutan)		2.33	ew	672	48
		Ringelblumenschnaps								0.91	ew	654	51
		Ringelblumentinktur								0.01	ew	367	54
		Ringelblumentinktur								0.01	ew	367	54
		Ringelblumentinktur								0.34	ew	219	32
		Ringelblumentinktur						exin (epicutan)		0.34	ew	219	32
		Ringelblumensalbe			oil/fat (et)	skin	cattle	exal (epicutan)		0.28	ew	220	32
		Ringelblumensalbe					cattle, calf	exal (epicutan)		1.82	od	436	62
		Ringelblumensalbe								0.28	ew	220	32
		Ringelblumensalbe						exin (epicutan)		0.28	ew	220	32
		Ringelblumensalbe					nsas	exal (epicutan)		3.27	od	274	35
		Ringelblumensalbe			oil/fat (rt)	musc	nsas	exin (epicutan)		1.73	ew	344	45
		Ringelblumensalbe				skin	cattle	exal (epicutan)		1.82	od	374	56
		Ringelblumensalbe								1.82	od	374	56
		Ringelblumensalbe								0.34	ew	276	36
		Ringelblumensalbe								0.34	ew	276	36
		Ringelblumenöl					cattle, calf	exal (epicutan)		2.22	ew	607	54
		Ringelblumensalbe								1.87	ew	394	57
		Ringelblumensalbe					nsas	exal (epicutan)		0.5	ew	587	59
		Ringelblumensalbe								1.52	ew	448	66
		Ringelblumentee			water (inf)	skin	nsas	exal (epicutan)		0.1	ew	389	59
		Ringelblumensalbe	pet	cu	oil/fat (rt)	skin	cattle	exal (epicutan)		1.22	ew	320	43
		Ringelblumensalbe					cattle, calf	exal (epicutan)		1.76	ew	563	68
		Ringelblumensalbe								1.22	ew	320	43
		Ringelblumensalbe					horse	exal (epicutan)		1.22	ew	320	43
		Ringelblumentee			water (inf)	gastr	calf	int (oral)	0.71		rd	319	43
		Ringelblumentee				skin	cattle	exal (epicutan)		0.2	rd	319	43
		Ringelblumentee								0.2	rd	319	43
		Ringelblumentee					horse	exal (epicutan)		0.2	rd	319	43
		Ringelblumentee					nsas	exal (epicutan)		0.2	rd	319	43
		Ringelblumentee					rabbit	exal (epicutan)		0.2	rd	319	43

Botanical family	Plant species	Recipe name designated by DP	Plant part	Origin	Extraction procedure on farm	Categories of use	Animal treated	Administration	Daily dosage [g/kg0.75]	Conc [g/100g]	Ver	RN	DP
**Asteraceae**	*Helianthus annuus* L.	Speiseöl	fsb	bo	none	mast	cattle	exin (epicutan)		na	na	202	30
		Speiseöl				para	cattle, calf	exin (epicutan)		na	na	202	30
		Speiseöl				skin	cattle, calf	exin (epicutan)		na	na	202	30
	Matricaria recutita L. (vs no : 71204)	Kamillentee	flo	bo	water (inf)	gastr	calf	int (oral)	5.88		od	380	56
		Kamillentee							0.79		od	697	47
		Kamillentee							0.35		od	674	48
		Kamillentee							0.16		od	651	51
		Kamillentee							0.53		ew	535	73
		Kamillentee							0.16		ew	512	71
		Kamillentee					cattle	int (oral)	1.94		od	380	56
		Kamillenspülung				infer	cattle	int (vaut)		0.75	od	652	51
		Kamillenspülung								0.3	ew	549	73
		Kamillentee								0.2	rd	206	30
		Kamillentee mit Soda								0.9	od	604	58
		Kamillentee				sens	young goat	exal (konj)		0.4	od	495	72
		Schmierseife und Kamillenbad				skin	cattle	exal (epicutan)		0.04	od	545	73
		Kamillenabsud					cattle, calf	exal (epicutan)		5	od	381	56
		Kamillentee								0.37	ew	564	68
		Kamillentee					nsas	exal (epicutan)		0.4	od	495	72
		Kamillosan®		bo*	none	skin	calf	exal (epicutan)		na	na	354	46
		Kamillosan®								na	na	257	34
		Kamillencreme		cu	oil/fat (rt)	skin	calf	exal (epicutan)		2.12	ew	661	50
		Kamillensalbe								5.65	ew	660	50
		Kamillencreme					cattle, calf	exal (epicutan)		2.12	ew	661	50
		Kamillensalbe								5.65	ew	660	50
		Kamillentee			water (inf)	gastr	calf	int (oral)	0.12		ew	397	57
		Kamillentee							2		rd	290	37
		Kamillentee							0.47		od	585	65
		Kamillentee					cattle	int (oral)	0.17		od	350	45
		Kamillentee				infer	cattle	int (vaut)		0.85	rd	290	37
		Kamillentee				skin	calf	exal (epicutan)		0.18	od	659	50
		Kamillentee					cattle	exal (epicutan)		0.85	rd	290	37
		Kamillentee								0.4	rd	445	64
		Kamillentee		wh	water (inf)	skin	cattle	exal (epicutan)		0.23	rd	428	61
		Kamillentee					nsas	exal (epicutan)		0.23	rd	428	61
	Matricaria recutita L. (vs no : 71204)	Kamillentee	na	bo	water (inf)	gastr	calf	int (oral)	0.68		od	335	44
		Kamillentee							1.32		od	335	44
	Senecio alpinus (L.) Scop (vs no: 71219)	Alpenkreuzkraut- Tee	her	wh	water (inf)	skin	cattle	exal (epicutan)		na	na	492	67
		Alpenkreuzkraut- Tee								na	na	472	69
		Buzlentee								na	na	419	60

Botanical family	Plant species	Recipe name designated by DP	Plant part	Origin	Extraction procedure on farm	Categories of use	Animal treated	Administration	Daily dosage [g/kg0.75]	Conc [g/100g]	Ver	RN	DP
**Asteraceae**	*Senecio ovatus* Willd.	Heidnisch Wundkraut	her	cu	water (inf)	skin	nsas	exal (epicutan)		na	na	640	52
		Heidnisch Wundkraut		wh	water (inf)	skin	cattle, calf	exal (epicutan)		na	na	425	61
		Heidnisch Wundkraut								na	na	382	56
		Heidnisch Wundkraut- Tee								na	na	680	48
		Heidnisch Wundkraut					nsas	exal (epicutan)		na	na	425	61
	Solidago virgaurea L.s.str.	Heidnisch Wundkraut- Salbe	her	bo*	none	skin	cattle	exal (epicutan)		na	na	646	51
		Heidnisch Wundkraut- Salbe								na	na	646	51
		Heidnisch Wundkraut- Salbe					cattle, calf	exal (epicutan)		na	na	646	51
		Goldrutencreme		wh	oil/fat (rt)	skin	cattle	exal (epicutan)		na	na	663	50
		Goldrutencreme								na	na	663	50
	Tanacetum parthenium (L.) Sch. Bip.	Mutterkraut	her	cu	none	varia	rabbit	int (oral)	na		na	331	43
	Tanacetum vulgare L.	Rainfarn- Tee	her	wh	water (inf)	para	cattle, calf	exin (epicutan)		na	na	458	66
**Betulaceae**	*Betula pendula* Roth	Birkenrinden- Tee	bar	bo	water (dec)	infer	cattle	int (oral)	na		na	601	58
**Boraginaceae**	*Symphytum officinale* L.	Beinwell	lea	cu	none	musc	cattle, calf	exin (epicutan)		da	na	261	34
	(vs-no: 71205)	Beinwell					goat	exin (epicutan)		da	na	261	34
		Wallwurz		wh	none	musc	nsas	exin (epicutan)		da	na	592	59
		Wallwurz- Emulsion	rob	bo*	none	musc	cattle, calf	exin (epicutan)		na	na	222	32
		Beinwell- Tinktur		cu	alcohol (rt)	skin	calf	exal (epicutan)		4.43	od	517	71
		Beinwell- Salbe			oil/fat (et)	musc	nsas	exin (epicutan)		6.35	od	352	45
		Wallwurz- Salbe				skin	nsas	exal (epicutan)		6.67	od	633	52
		Wallwurz- Salbe			oil/fat (rt)	musc	cattle	exin (epicutan)		1.82	od	383	56
		Wallwurz- Salbe					cattle, calf	exin (epicutan)		3.45	ew	562	68
		Beinwell- Tinktur		wh	alcohol (rt)	mast	cattle	exin (epicutan)		8.33	ew	270	35
		Beinwell- Tinktur				musc	cattle	exin (epicutan)		8.33	ew	270	35
		Wallwurz- Schnaps								3.51	ew	657	51
		Beinwell- Tinktur				skin	nsas	exal (epicutan)		4.26	ew	581	65
		Beinwell			none	musc	cattle	exin (epicutan)		da	na	706	47
		Wallwurz					cattle, calf	exin (epicutan)		da	na	656	51
		Wallwurz					nsas	exin (epicutan)		da	na	591	59
		Beinwell				skin	nsas	exal (epicutan)		da	na	580	65
**Brassicaceae**	*Brassica napus* L.	Rapsöl& Schnaps	fsb	bo	none	gastr	calf	int (oral)	7.84		od	602	58
		Rapsöl& Schnaps					cattle	int (oral)	0.78		od	602	58
		Rapsöl				para	calf	exin (epicutan)		da	na	283	36
		Schwefelblüten& Rapsöl				skin	cattle, calf	exal (epicutan)		da	na	702	47
	Brassica oleracea L.	Kabisblatt	lea	cu	none	musc	cattle	exin (epicutan)		da	na	271	35
		Kohl					cattle, calf	exin (epicutan)		da	na	658	51
		Kohl				skin	cattle	exal (epicutan)		da	na	658	51
		Kohl								da	na	265	34
		Kohl					nsas	exal (epicutan)		da	na	634	52
	Capsella bursa- pastoris (L.) Medik	Hirtentäschli- Tee	her	wh	water (rt)	varia	cattle	int (oral)	0.05		rd	489	67

Botanical family	Plant species	Recipe name designated by DP	Plant part	Origin	Extraction procedure on farm	Categories of use	Animal treated	Administration	Daily dosage [g/kg0.75]	Conc [g/100g]	Ver	RN	DP
**Cannabaceae**	*Cannabis sativa* L.	Futterhanf	her	bo	none	streng	cattle	int (oral)	na		na	650	51
**Chenopodiaceae**	*Chenopodium bonus-henricus* L.	Wundsalbe	lea	wh	oil/fat (rt)	skin	nsas	exal (epicutan)		na	na	456	66
**Cupressaceae**	*Juniperus communis* L.s.str	Wacholdersalbe	twb	wh	alcohol (rt)	mast	cattle	exal (epicutan)		na	na	395	57
		Wacholdersalbe			alcohol (rt)	musc	cattle	exin (epicutan)		na	na	395	57
		Wachholder			none	streng	cattle, calf	tohe	tohe	tohe	na	453	66
		Wachholder							tohe	tohe	na	453	66
	Thuja occidentalis L.	Thuja- Tee	twb	cu	water (inf)	infer	cattle	int (oral)	0.01		ew	409	57
		Thuja- Tee		wh	water (inf)	infer	cattle	int (oral)	na		na	558	73
**Equisetaceae**	*Equisetum arvense* L.	Katzenschwanz- Tee	her	wh	water (inf)	gastr	calf	int (oral)	0.07		ew	642	52
**Ericaceae**	*Vaccinium myrtillus* L.	Heidelbeersaft	fsb	wh	water (dec)	gastr	calf	int (oral)	na		na	511	71
		Heidelbeerstauden- Tee	twb	wh	water (dec)	gastr	calf	int (oral)	0.47		ew	681	48
**Fabaceae**	*Anthyllis vulneraria* L.s.str.	Wundklee	flo	wh	water (inf)	skin	cattle	exal (epicutan)		na	na	431	61
		Eichenrindenpulver	bar	bo	none	gastr	calf	int (oral)	0.78		od	372	55
		Eichenrindentee		wh	water (dec)	gastr	calf	int (oral)	0.08		rd	474	69
		Eichenrindentee					cattle	int (oral)	0.05		od	405	57
		Eichenrindentee				infer	cattle	int (vaut)		0.8	od	678	48
		Eichenrinde				skin	cattle	exal (epicutan)		na	na	444	64
**Gentianaceae**	*Gentiana lutea* L.	Enzianschnaps	rob	wh	alcohol (rt)	gastr	calf	int (oral)	0.03		ew	476	69
**Geraniaceae**	*Geranium robertianum* L.s.str.	Storchenschnabel	her	bo	none	infer	cattle	int (oral)	0.11		od	465	69
	(vs no: 71213, 71220)	Storchenschnabelsalbe		cu	oil/fat (rt)	skin	nsas	exal (epicutan)		0.9	ew	451	66
		Storchenschnabeltinktur		wh	alcohol (rt)	behav	cattle	int (oral)	0.001		ew	396	57
		Storchenschnabel			none	infer	cattle	int (oral)	0.02		rd	641	52
		Storchenschnabelcreme			oil/fat (et)	skin	cattle, calf	exal (epicutan)		0.94	od	516	71
		Storchenschnabelsalbe					cattle	exal (epicutan)		0.74	ew	568	68
		Storchenschnabelsalbe					cattle, calf	exal (epicutan)		0.74	ew	568	68
		Storchenschnabelsalbe								0.74	ew	568	68
		Storchenschnabelsalbe					dog	exal (epicutan)		0.71	ew	328	43
	Pelargonium sidoides DC	Pelargo®	rob	bo*	none	resp	calf	int (oral)	na		na	254	34

Botanical family	Plant species	Recipe name designated by DP	Plant part	Origin	Extraction procedure on farm	Categories of use	Animal treated	Administration	Daily dosage [g/kg0.75]	Conc [g/100g]	Ver	RN	DP
**Hypericaceae**	*Hypericum perforatum* L.	Johanniskraut- Öl	flo	bo*	none	musc	cattle	exin (epicutan)		na	na	497	72
	(vs no: 71202, 71216, 71223)	Johannisöl				skin	calf	exal (epicutan)		na	na	596	58
		Johannisöl						exin (epicutan)		na	na	596	58
		Johannisöl					cattle	exal (epicutan)		na	na	596	58
		Johannisöl		cu	oil/fat (rt)	infer	cattle	int (vaut)		0.76	ew	387	57
		Johannisöl				musc	nsas	exin (epicutan)		1.49	ew	346	45
		Johannisöl				skin	calf	exin (epicutan)		0.76	ew	387	57
		Johannisöl					cattle	exal (epicutan)		2	ew	612	54
		Johannissalbe								0.66	ew	390	57
		Johannisöl					cattle, calf	exal (epicutan)		2	ew	612	54
		Johannisöl								2	ew	612	54
		Johannisöl								2	od	376	56
		Johannisöl		wh	oil/fat (rt)	infer	cattle	int (vaut)		3	od	518	71
		Johannisöl				mast	cattle	exin (epicutan)		3	od	518	71
		Johannistinktur				musc	cattle	exin (epicutan)		1.89	ew	427	61
		Johanniskraut in Olivenöl					nsas	exin (epicutan)		1.43	ew	262	34
		Johanniskraut- Öl				skin	cattle	exal (epicutan)		1.96	ew	481	69
		Johannisöl								3	od	518	71
		Johanniskraut- Öl					cattle, calf	exal (epicutan)		1.96	ew	481	69
		Johannisöl								0.62	ew	590	59
		Johannistinktur								1.89	ew	427	61
**Juglandaceae**	*Juglans regia* L.	Nussbaumblätter	lea	wh	none	para	calf	tohe	tohe	tohe	na	653	51
**Lamiaceae**	*Lavandula angustifolia* Mill.	Lavendelsträusschen	her	cu	none	para	nsas	tohe	tohe	tohe	na	332	43
		Lavendelöl			oil/fat (rt)	skin	cattle, calf	exal (epicutan)		9.09	ew	613	54
		Lavendelöl				streng	cattle, calf	tohe	tohe	tohe	ew	613	54
	Mentha canadensis L.	NPJ Liniment®	lea	bo*	none	mast	cattle	exin (epicutan)		na	na	296	37
		OPIFIX®								na	na	339	44
		OPIFIX®				musc	cattle	exin (epicutan)		na	na	339	44
		Pfefferminztee	na	bo	water (inf)	gastr	calf	int (oral)	0.62		od	334	44
		Pfefferminztee							1.24		od	334	44
	Salvia officinalis L.	Salbei	lea	cu	none	resp	calf	int (oral)	0.02		ew	285	36
		Salbei				skin	rabbit	int (oral)	na		na	329	43
		Salbeitee	lea	cu	water (inf)	gastr	calf	int (oral)	0.02		ew	636	52
	Thymus vulgaris L.	ätherisches Öl Thymian	her	bo*	none	resp	calf	int (nasal)	na		na	253	34
**Lauraceae**	*Cinnamomum verum* J.Presl	Zimt	bar	bo	none	gastr	calf	int (oral)	0.24		ew	553	73

Botanical family	Plant species	Recipe name designated by DP	Plant part	Origin	Extraction procedure on farm	Categories of use	Animal treated	Administration	Daily dosage [g/kg0.75]	Conc [g/100g]	Ver	RN	DP
**Linaceae**	*Linum usitatissimum* L.	Leinsamen	fsb	bo	none	infer	cattle	int (oral)	0.62		ew	599	58
		Leinsamen				streng	cattle	int (oral)	1.57		od	309	39
		Leinsamenschleim			water (dec)	gastr	calf	int (oral)	4.21		rd	584	65
		Leinsamenschleim							4.21		rd	584	65
		Leinsamenschleim							15.69		od	527	70
		Leinsamentee							10.97		od	214	31
		Leinsamen					cattle	int (oral)	0.78		od	508	71
		Leinsamenmus							7.78		od	379	56
		Leinsamenschleim							7.78		od	700	47
		Leinsamenschleim							1.62		rd	584	65
		Leinsamenschleim							1.62		rd	584	65
		Leinsamenschleimspülung				infer	cattle	int (vaut)		2.22	rd	242	33
		Leinsamenschleim				musc	cattle	exin (epicutan)		20	od	310	39
**Lycopodiaceae**	*Lycopodium clavatum* L.	Bärlapp	her	wh	none	musc	cattle, calf	tohe	tohe	tohe	na	204	30
**Malvaceae**	*Althaea officinalis* L.	Eibischblätter	lea	wh	water (inf)	skin	cattle	exal (epicutan)		na	na	392	57
	Malva neglecta Wallr. (vs no: 71212)	Chäslichrut	flo	wh	water (inf)	skin	cattle, calf	exal (epicutan)		0.1	ew	430	61
		Chäslichrut	her	bo	water (inf)	skin	cattle	exal (epicutan)		0.4	rd	499	72
		Malventee			water (inf)	skin	cattle	exal (epicutan)		0.2	od	323	43
		Malventee			water (inf)	skin	horse	exal (epicutan)		0.2	od	323	43
		Chäslichrut- Salbe		cu	oil/fat (rt)	skin	cattle	exal (epicutan)		1.82	od	375	56
		Chäslichrut- Salbe								1.82	od	375	56
		Chäslichrut - Salbe					cattle, calf	exal (epicutan)		na	na	566	68
		Chäslichrut			water (inf)	skin	cattle	exal (epicutan)		na	na	662	50
		Chäslichrut - Tee					cattle, calf	exal (epicutan)		0.4	od	565	68
		Chäslichrut - salbe		wh	oil/fat (et)	musc	cattle, calf	exin (epicutan)		1.82	od	434	62
		Chäslichrut - Salbe			oil/fat (et)	skin	cattle	exal (epicutan)		1.82	od	434	62
		Chäslichrut - Bad			water (inf)	skin	calf	exal (epicutan)		na	na	433	62
		Chäslichrut - Tee								na	na	696	47
		Chäslichrut					cattle	exal (epicutan)		0.8	od	298	37
		Chäslichrut								0.8	od	298	37
		Chäslichrut - Tee								na	na	696	47
		Chäslichrut- Bad								na	na	433	62
		Chäslichrut- Bad								na	na	433	62
		Chäslichrut - Bad					cattle, calf	exal (epicutan)		na	na	217	31
		Chäslichrut - Tee					horse	exal (epicutan)		na	na	696	47
		Chäslichrut - Tee			water (rt)	skin	cattle	exal (epicutan)		na	na	635	52
		Chäslichrut - Tee				skin	nsas	exal (epicutan)		na	na	635	52
	Tilia cordata Mill.	Lindenbast	bar	bo	none	infer	cattle	int (oral)	0.19		od	373	55
		Lindenbast			water (dec)	infer	cattle	int (oral)	0.13		od	414	60
		Lindenbast- Tee							na		na	600	58
		Lindenrinden- Tee		wh	water (dec)	infer	cattle	int (oral)	na		na	677	48
		Lindenbast						int (vaut)		na	na	252	33
**Myristicaceae**	*Myristica fragrans* Houtt.	Muskatnuss	fsb	bo	none	gastr	cattle	int (oral)	0.05		ew	408	57
		Muskatnuss							0.05		ew	236	33

Botanical family	Plant species	Recipe name designated by DP	Plant part	Origin	Extraction procedure on farm	Categories of use	Animal treated	Administration	Daily dosage [g/kg0.75]	Conc [g/100g]	Ver	RN	DP
**Myrtaceae**	*Melaleuca alternifolia*	Teebaumöl	lea	bo*	none	skin	nsas	exal (epicutan)		na	na	255	34
	Maiden&Betche ex Cheel	Teebaumöl				streng	nsas	tohe	tohe	tohe	na	255	34
		Teebaumöl	na	bo*	none	mast	cattle	exin (epicutan)		na	na	340	44
**Oleaceae**	*Fraxinus excelsior* L.	Eschenlaub	lea	wh	none	para	calf	int (oral)	na		na	473	69
		Esche	twb	wh	none	streng	cattle	int (oral)	na		na	463	66
	Olea europaea L.	Olivenöl und Eigelb	fsb	bo	none	mast	cattle	exin (epicutan)		da	na	406	57
**Orobanchaceae**	*Pedicularis verticillata* L.	Läusekraut- Tee	her	wh	water (inf)	para	cattle, calf	exin (epicutan)		na	na	459	66
**Pinaceae**	*Abies alba* Mill.	Weisstannen- Äste	twb	wh	none	streng	cattle, calf	int (oral)	na		na	683	48
	Picea abies (L.) H. Karst.	Harzsalbe	exc	bo	oil/fat (et)	mast	cattle	exin (epicutan)		na	na	377	56
		Harzsalbe				resp	calf	exin (epicutan)		na	na	377	56
		Harzsalbe				skin	cattle	exal (epicutan)		na	na	377	56
		Tannenharz		wh	none	skin	cattle, calf	exal (epicutan)		da	na	478	69
		Harzsalbe			oil/fat (et)	mast	cattle	exin (epicutan)		3.53	od	515	71
		Harzsalbe								27.27	od	432	62
		Harzsalbe				resp	calf	exin (epicutan)		27.27	od	432	62
		Harzsalbe				skin	calf	exal (epicutan)		27.27	od	432	62
		Harzsalbe					cattle	exal (epicutan)		3.53	od	515	71
		Harzsalbe								27.27	od	432	62
		Harzsalbe								27.27	od	432	62
		Harzsalbe								31.75	od	343	45
		Harzsalbe						int (oral)	na		na	432	62
		Harzsalbe					cattle, calf	exal (epicutan)		na	na	480	69
		Harzsalbe								na	na	480	69
		Harzsalbe								27.27	od	432	62
		Harzsalbe								27.27	od	432	62
		Harzsalbe								12.93	od	393	57
		Harzsalbe					nsas	exal (epicutan)		31.75	od	343	45
		Rottannen- Äste	twb	wh	none	resp	calf	int (oral)	na		na	645	51
		Rottannen- Zweige							na		na	288	36
		Tannenäste							na		na	484	69
		Tannenäste							na		na	440	62
		Rottannen- Äste				streng	cattle, calf	int (oral)	na		na	682	48
**Plantaginaceae**	*Euphrasia officinalis* L.	Augentrost	her	wh	water (inf)	sens	cattle, calf	exal (konj)		1	od	461	66
	Plantago lanceolata L. (vs no: 71211)	Spitzwegerich	lea	wh	none	resp	calf	int (oral)	0.12		ew	280	36
		Spitzwegerich-Öl			oil/fat (rt)	mast	cattle	exin (epicutan)		0.32	ew	326	43
**Poaceae**	*Avena sativa* L.s.str.	Haferschleimsuppe	fsb	bo	water (dec)	gastr	calf	int (oral)	5.6		ew	503	72
		Hafer		cu	none	streng	cattle	int (oral)	0.31		ew	227	33
		Haferwickel			water (dec)	resp	calf	exin (epicutan)		na	na	266	34

Botanical family	Plant species	Recipe name designated by DP	Plant part	Origin	Extraction procedure on farm	Categories of use	Animal treated	Administration	Daily dosage [g/kg0.75]	Conc [g/100g]	Ver	RN	DP
**Polygonaceae**	*Rumex obtusifolius* L.	Blacke	lea	wh	none	musc	cattle, calf	exin (epicutan)		da	na	260	34
	(vs no: 71208, 71218)	Blacke					goat	exin (epicutan)		da	na	260	34
		Blacke				skin	horse	exin (epicutan)		da	na	325	43
		Blacke					nsas	exal (epicutan)		da	na	424	61
		Blackensalbe			oil/fat (et)	mast	cattle	exin (epicutan)		3.64	od	514	71
		Blackensalbe								1.82	od	435	62
		Blackensalbe								3.08	od	215	31
		Blackensalbe				musc	cattle	exin (epicutan)		20	od	403	57
		Blackensalbe					cattle, calf	exin (epicutan)		3.08	od	215	31
		Blackensalbe				skin	cattle	exal (epicutan)		2.77	od	275	35
		Blackensalbe					cattle, calf	exal (epicutan)		3.59	ew	648	51
		Blackensalbe								1.82	od	435	62
		Blackensalbe					nsas	exal (epicutan)		2.77	od	275	35
		Blackensalbe				varia	cattle	exin (epicutan)		3.64	od	514	71
		Blackensalbe			oil/fat (rt)	mast	cattle	exin (epicutan)		10	od	631	52
		Blackensalbe								1.98	ew	561	68
		Blackensalbe								1.52	ew	483	69
		Blackensalbe				musc	cattle	exin (epicutan)		1.98	ew	561	68
		Blackensalbe					cattle, calf	exin (epicutan)		1.52	ew	483	69
		Blackensalbe								1.52	ew	460	66
		Blackensalbe				skin	cattle	exal (epicutan)		1.75	ew	324	43
		Blackensalbe					cattle, calf	exal (epicutan)		1.52	ew	483	69
		Blackensalbe					nsas	exal (epicutan)		10	od	631	52
		Blackenöl				varia	cattle	exin (epicutan)		2.33	ew	614	54
		Blackensalbe								2	ew	611	54
		Blackensalbe								1.98	ew	561	68
		Blackenwurzel- Tee	rob	wh	water (dec)	gastr	calf	int (oral)	0.82		ew	348	45
		Blackenwurzel- Tee							1.18		ew	207	30
		Blackenwurzel-Tee							3.15		od	355	46
		Blackentee				skin	cattle, calf	exal (epicutan)		0.73	ew	583	65
		Blackentee	wpr	wh	water (inf)	gastr	calf	int (oral)	0.78		ew	423	61
		Blackentee					young pig	int (oral)	1.32		ew	423	61
**Rhamnaceae**	*Rhamnus cathartica* L.	Kreuzdorn	twb	wh	none	skin	cattle, calf	tohe	tohe	tohe	na	468	69
		Kreuzdorn							tohe	tohe	na	359	46
		Kreuzdorn							tohe	tohe	na	286	36
**Rosaceae**	*Alchemilla vulgaris* L. Agg.	Frauenmänteli- Tee	her	wh	water (inf)	infer	cattle	int (oral)	0.03		ew	488	67
		Frauenmänteli- Tee							0.03		ew	488	67
	Crataegus laevigata (Poiret) DC.	Weissdorn	twb	wh	none	skin	cattle, calf	tohe	tohe	tohe	na	504	72
		Weissdorn							tohe	tohe	na	466	69
		Weissdorn							tohe	tohe	na	314	39
		Weissdorn							tohe	tohe	na	287	36
	Malus domesticus Borkh.	Apfel	fruits	cu	none	gastr	cattle	int (oral)	na		na	233	33

Botanical family	Plant species	Recipe name designated by DP	Plant part	Origin	Extraction procedure on farm	Categories of use	Animal treated	Administration	Daily dosage [g/kg0.75]	Conc [g/100g]	Ver	RN	DP
**Rosaceae**	*Potentilla erecta* (L.) Raeusch.	Blutwurz- Tinktur	na	bo*	none	gastr	calf	int (oral)	na		na	224	32
	(vs no: 71201)	Blutwurz	rob	bo	water (dec)	gastr	cattle	int (oral)	0.11		od	507	71
		Blutwurz- Tee							0.44		od	213	31
		Blutwurz- Tinktur		wh	alcohol (rt)	gastr	calf	int (oral)	0.004		ew	477	69
		Blutwurz- Tinktur				streng	calf	int (oral)	0.004		ew	477	69
	Prunus domestica L.	Zwetschgensteine	fsb	cu	none	para	pig	int (oral)	na		na	250	33
	Prunus spinosa L.	Schwarzdorn	twb	wh	none	skin	cattle, calf	tohe	tohe	tohe	na	586	65
		Schwarzdorn							tohe	tohe	na	556	73
		Schwarzdorn							tohe	tohe	na	467	69
	Rubus idaeus L.	Himbeerstauden	twb	wh	none	streng	cattle	int (oral)	na		na	462	66
**Rubiaceae**	*Coffea* L.	Kaffee- Schnaps	fsb	bo	water (inf)	gastr	calf	int (oral)	0.04		ew	593	59
		Kaffee- Schnaps					cattle	int (oral)	0.45		od	684	46
		Kaffee- Schnaps							0.51		ew	578	65
		Kaffee- Schnaps							0.06		ew	573	68
		Kaffee- Schnaps							1.06		ew	543	73
		Kaffee- Schnaps							0.13		od	519	71
		Kaffee- Schnaps							0.09		rd	500	72
		Kaffee- Schnaps							0.71		ew	494	70
		Kaffee- Schnaps							0.07		od	439	62
		Kaffee- Schnaps							0.19		od	413	60
		Kaffee- Schnaps							0.19		od	413	60
		Kaffee- Schnaps							0.04		od	398	57
		Kaffee- Schnaps							0.45		od	360	47
		Kaffee- Schnaps							0.31		od	303	39
		Kaffee- Schnaps							0.31		od	294	37
		schwarzer Kaffee					rabbit	int (oral)	1.58		od	317	40
		Kaffee- Schnaps					young sheep	int (oral)	0.11		ew	593	59
		Kaffee- Schnaps				infer	cattle	int (oral)	0.06		od	211	30
**Rutaceae**	*Citrus x limon* (L.) Burm.f.	Zitronensaft	fsb	bo	none	gastr	calf	int (oral)	0.47		od	416	60
**Salicaceae**	*Salix caprea* L.	Salweidenzweige	twb	wh	none	gastr	cattle	int (oral)	na		na	235	33
**Simaroubaceae**	*Quassia amara* L.	Fliegenholz	bar	bo	alcohol (rt)	skin	donkey	exal (epicutan)		na	na	315	40
**Solanaceae**	*Solanum tuberosum* L.	Kartoffelsalbe	rob	cu	oil/fat (et)	skin	cattle	exal (epicutan)		5	ew	401	57
		Kartoffelsalbe								5	ew	401	57
		Kartoffelsalbe			oil/fat (rt)	skin	cattle	exal (epicutan)		1.72	ew	402	57
		Kartoffelsalbe								1.72	ew	402	57
**Theaceae**	*Camellia sinensis* (L.) Kuntze	Schwarztee	lea	bo	water (inf)	gastr	calf	int (oral)	0.53		ew	536	73
		Schwarztee							0.56		rd	446	64
		Schwarztee							0.12		od	404	57
		Schwarztee							1.74		ew	336	44
		Schwarztee							0.71		ew	322	43
		Schwarztee							0.62		od	273	35
		Schwarztee					dog	int (oral)	0.18		od	273	35
**Tropaeolaceae**	*Tropaeolum majus* L.	Kapuzinerkresse	flo	wh	none	streng	horse	int (oral)	na		na	330	43

Botanical family	Plant species	Recipe name designated by DP	Plant part	Origin	Extraction procedure on farm	Categories of use	Animal treated	Administration	Daily dosage [g/kg0.75]	Conc [g/100g]	Ver	RN	DP
**Urticaceae**	*Urtica dioica* L.	Brennnessel	her	wh	none	behav	pig	int (oral)	1.05		ew	399	57
	(vs no: 71209, 71210, 71217)	Brennnessel				gastr	cattle	int (oral)	na		na	238	33
		Brennnessel					donkey	int (oral)	0.79		ew	316	40
		Brennnessel					goat	int (oral)	na		na	365	52
		Brennnessel					sheep	int (oral)	na		na	365	52
		Brennnessel				infer	cattle	int (oral)	0.02		ew	447	65
		Brennnessel						int (oral)	0.03		ew	216	31
		Brennnessel				skin	donkey	int (oral)	na		na	316	40
		Brennnessel				streng	cattle	int (oral)	na		na	485	69
		Brennnessel							na		na	347	45
		Brennnessel							na		na	258	34
		Brennnessel					goat	int (oral)	0.9		ew	411	61
		Brennnessel							na		na	258	34
		Brennnessel					hen	int (oral)	na		na	347	45
		Brennnessel					horse	int (oral)	na		na	258	34
		Brennnesseln				Varia	cattle	int (oral)	0.26		ew	649	51
		Brennnessel- Tee			water (inf)	gastr	calf	int (oral)	0.55		ew	630	52
		Brennnessel- Tee							2.21		ew	388	58
		Brennnessel- Tee					cattle	int (oral)	0.22		ew	630	52
		Brennnessel- Tee							0.96		od	386	56
		Brennnessel				infer	cattle	int (oral)	0.16		ew	498	72
		Brennnessel- Tee							0.1		ew	259	34
		Brennnessel- Tee							0.02		ew	575	65
		Brennnessel- Tee							0.22		ew	531	73
		Brennnessel- Tee							0.26		ew	363	50
		Brennnessel- Tee					goat	int (oral)	0.53		ew	259	34
		Brennnessel- Tee					pig	int (oral)	0.06		ew	575	65
		Brennnessel- Tee				skin	nsas	exal (epicutan)		0.24	ew	422	61
		Brennnessel- Tee				streng	cattle	int (oral)	0.43		ew	595	58
